# Fine‐Tuned Regulation of mRNA Translation and Transport by STAU2 Condensate Facilitates Neuronal Development and Plasticity

**DOI:** 10.1002/advs.202600044

**Published:** 2026-05-29

**Authors:** Shijing Huang, Yuanyuan Chu, Ruiqian Zhao, Siyu Li, Yu Feng, Hongdan Zheng, Yunli Xie, Tong Wang, Wenyu Wen

**Affiliations:** ^1^ Department of Neurosurgery Huashan Hospital the Shanghai Key Laboratory of Medical Epigenetics State Key Laboratory of Brain Function and Disorders and MOE Frontiers Center for Brain Science Institutes of Biomedical Sciences Shanghai Medical College Fudan University Shanghai China; ^2^ School of Life Science and Technology ShanghaiTech University Shanghai China; ^3^ Institutes of Brain Science Fudan University Shanghai China

**Keywords:** dendritic development, phase separation, RNA translation, RNA transport, STAU2

## Abstract

The double‐stranded RNA‐binding protein STAU2 plays an essential role in neural development and synaptic plasticity, assembling target mRNAs into ribonucleoprotein (RNP) granules to control their trafficking, localization, and local translation, though the detailed molecular mechanism remains elusive. Here, we show that STAU2 phase separates to form dynamic condensates in dendrites of hippocampal neurons, recruiting specific mRNAs to assemble mobile RNP granules that are transported distally along microtubules. These RNA‐loaded STAU2 condensates undergo a liquid‐to‐gel transition, which stabilizes the encapsulated transcripts while repressing their translation. During neuron development, disrupting STAU2 condensation impairs RNP formation and compromises anterograde mRNA delivery to distal dendrites. Conversely, STAU2 overexpression promotes excessive coacervation, resulting in oversized RNP granules with reduced mobility, ultimately hindering dendritic elongation and promoting excessive branching. We further demonstrate that synaptic activity bidirectionally remodels STAU2 condensates in parallel with changes in local translation of bound mRNAs. Notably, aberrant STAU2 condensates emerge as a potential pathological feature in aggregation‐prone neurodegenerative disorders. Collectively, our findings establish that STAU2 condensate safeguards the development of postmitotic neurons by orchestrating the dendritic transport and activity‐dependent translation of its target mRNAs.

## Graphical abstract

STAU2 undergoes phase separation to form dynamic condensates that package target mRNAs and deliver them to the distal ends of growing neuronal dendrites. STAU2 condensates stabilize embedded mRNAs and repress their translation. Synaptic activity bidirectionally remodels STAU2 condensates, coordinating local translation of STAU2‐associated mRNAs. Dysregulated STAU2 condensation impairs dendritic development and may be associated with neurodegeneration.

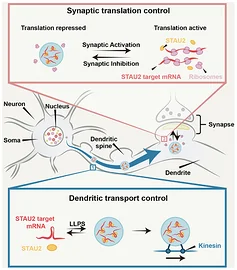

## 1 Introduction

mRNAs exhibit distinct subcellular localizations in various cell types, where their targeted transport and local translation are essential for fundamental processes including asymmetric cell division, neurogenesis, and synaptic plasticity [1, 2]. This spatial regulation is driven by the dynamic co‐assembly of two key biopolymers, mRNAs and RNA‐binding proteins (RBPs), into membraneless ribonucleoprotein (RNP) granules. Through specific polymer‐polymer interactions, these RNPs not only guide mRNAs to precise subcellular locations but also enable their spatiotemporal translation, thereby ensuring precisely tuned gene expression critical for specialized cellular functions.

Staufen, a double‐stranded RNA (dsRNA)‐binding protein evolutionarily conserved from *Drosophila* to mammals, stands as a master regulator in the realm of mRNA localization, stability, translation, and RNP biogenesis [3, 4, 5, 6, 7]. Initially recognized for its role in orchestrating *oskar* and *bicoid* mRNA localization in *Drosophila* oocytes [8, 9], Staufen functioned as a cell fate determinant in early neurogenesis. It preferentially associates with mRNAs *prospero* and brain tumor (*brat*) at the basal cortex of asymmetrically dividing neuroblasts, ensuring their inheritance by basal ganglion mother cell (GMC) to initiate differentiation, thereby sculpting neural fate [3, 10, 11, 12]. In mammals, two Staufen orthologs, STAU1 and STAU2, have evolved to steer distinct physiological pathways through the assembly of specialized RNPs via their conserved RNA‐binding domains (RBDs) [13, 14]. STAU1 is ubiquitously expressed, whereas STAU2 is predominantly expressed in the brain and heart [15, 16]. Echoing its *Drosophila* counterpart, STAU2 plays a pivotal role in mammalian neurogenesis by directing the asymmetric localization and segregation of *PROX1* mRNA (mammalian homologue of *prospero*) and *TRIM32* mRNA (mammalian homologue of *brat*) during asymmetric neural stem divisions [11, 17].

Besides neurogenesis, STAU2 also plays essential roles in orchestrating synaptic development and plasticity in postmitotic neurons [18, 19]. In neurons, STAU2 assembles target mRNAs into RNPs and maintains them in a translationally repressed state [20]. These STAU2 RNPs navigate along microtubules toward dendritic spines, where synaptic activity is linked to local translation of dendritic mRNAs involved in shaping synaptic plasticity [3, 14, 21, 22, 23, 24, 25]. STAU2 knockdown (KD) results in diminished dendritic spine maturity and reduced synaptic currents in cultured hippocampal neurons [18], and hindered mGluR‐dependent long‐term depression (LTD) in organotypic hippocampal slice cultures [14]. STAU2 deficiency in rats has been linked to cognitive impairments, underscoring its centrality to learning and memory [19]. Recent explorations have mapped nearly 8000 genes within STAU2 granules across four stages of mouse cortical development, implicating STAU2 in a vast array of cellular processes from chromosomal organization to RNA transport, through temporally orchestrated gene assembly [11, 14, 18, 19, 21, 26]. Remarkably, even at a single developmental juncture like embryonic day 17.5, STAU2 forms granules with a multitude of RNAs, many of which are integral to synaptic development and plasticity, including G‐protein signaling regulator 4 (*RGS4*), calcium/calmodulin‐dependent protein kinase II α (*CaMKIIα*), microtubule‐associated protein 1B (*Map1b*) and β‐actin *(Actb*) [14, 18, 27, 28, 29]. Yet, despite all these critical functions in the central nervous system, the mechanisms by which STAU2 co‐packages diverse mRNAs into RNP granules and coordinates their trafficking and translational control across cortical development and activity‐dependent states remain veiled. Unraveling these mysteries stands to illuminate not just the mechanistic basis of STAU2 in neuronal development and plasticity, but also the broader principles governing RNA metabolism in health and disease.

In this study, we explored the organizational principles of STAU2 granules and their roles in controlling target RNA translation and trafficking during dendritic maturation, using primary hippocampal neurons dissociated from E17 rat embryos. Our data reveal that STAU2 forms dynamic granules in neuronal dendrites to deliver target mRNAs along microtubules via liquid‐liquid phase separation (LLPS) [30, 31, 32, 33, 34, 35]. RNA recruitment induces gelation of STAU2 condensates, which suppresses the translation of encapsulated mRNAs in a reversible manner. This translationally restrained state is bidirectionally remodeled by synaptic activity. In dendrites of hippocampal neurons, either insufficient or excessive STAU2 phase separation disrupts long‐distance mRNA transport. These findings underscore the pivotal role of balanced phase separation of STAU2 polymers in spatially governing mRNA translation and trafficking—a process fundamental to the development and plasticity of the neural network.

## 2 Results

### 2.1 STAU2 is Expressed in Neurons Throughout the Mouse Brain

**FIGURE 1:**
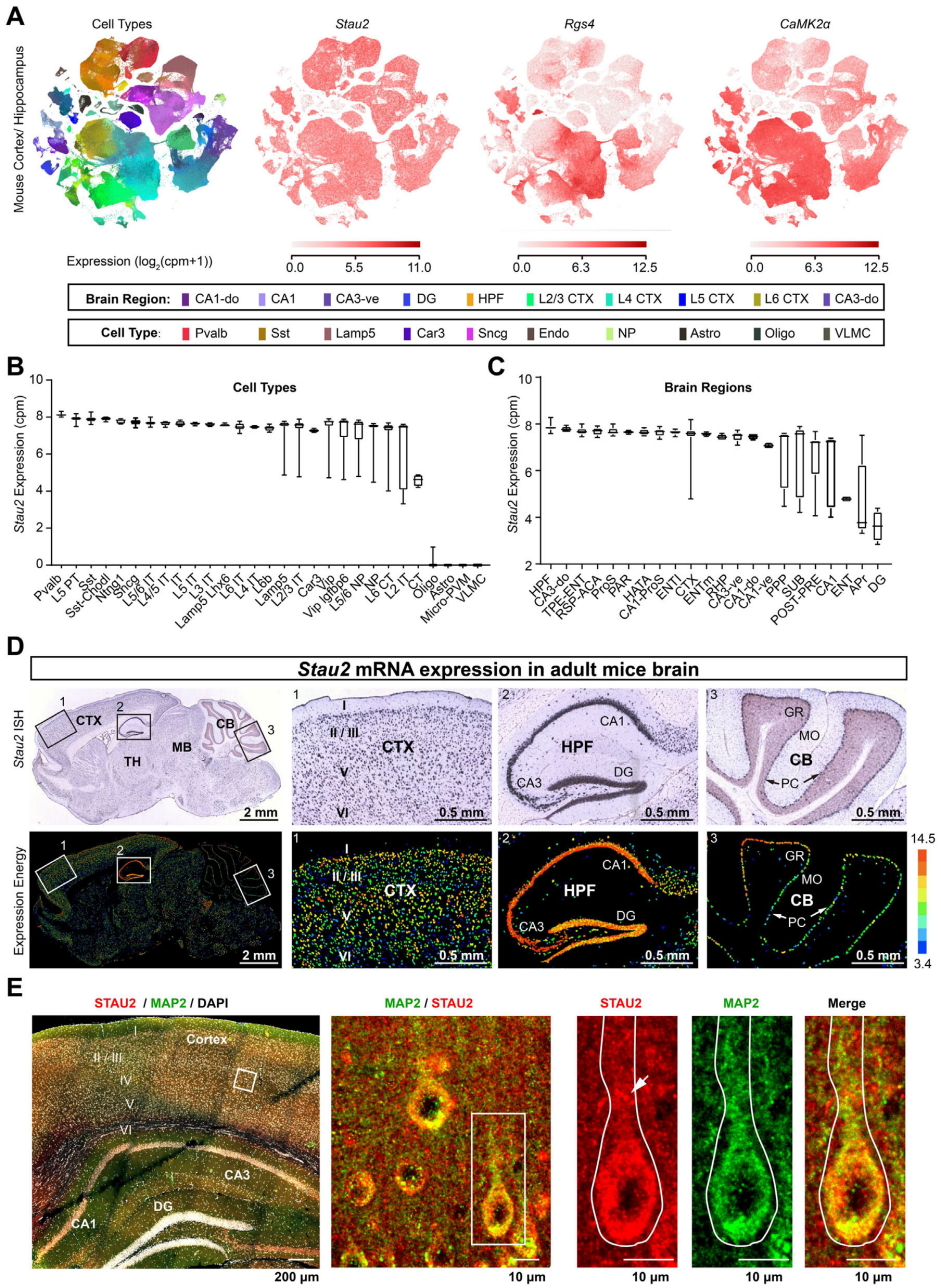
STAU2 is expressed in neurons throughout the mouse brain. (A) Analysis of single‐cell RNA sequencing data (Allen Institute for Brain Science) from sorted mouse cortical and hippocampal neurons for expression levels of *STAU2*, *RGS4* and *CaMK2α*. (B‐C) *STAU2* expression across different cell types (B) and brain regions (C) in sorted mouse cortical and hippocampal neurons, based on RNA‐seq data from the Allen Institute for Brain Science. (D) Representative images of STAU2 in situ hybridization (ISH) and corresponding expression energy maps in the adult mouse brain (Allen Brain Atlas). Boxed regions in the cortex (CTX), hippocampus (HPF), and cerebellum (CB) are magnified in the right panels. Scale bars, 2 mm (left), 0.5 mm (right). (E) Representative IF images of coronal sections from a 4‐month‐old mouse brain showing STAU2 (red), the dendritic marker MAP2 (green), and nuclei (DAPI, gray). Higher‐magnification views of layer 3/4 cortical neurons are shown in the right panels. Arrows indicate STAU2 granules in neuronal dendrites. Scale bars: 200 µm (left), 10 µm (middle and right).

To assess the physiological relevance of STAU2 in vivo, we analyzed publicly available Allen Institute transcriptomic datasets **(reference no**. 248566). UMAP visualization and cell‐type quantification showed that *STAU2* mRNA is broadly enriched in neuronal populations, including excitatory neurons, inhibitory neurons, and cerebellar Purkinje cells, whereas its expression is relatively low in most non‐neuronal cell types (Figure 1A,B). Region‐level analysis further revealed prominent STAU2 expression in multiple brain areas, including the cerebral cortex, hippocampal formation, and cerebellum (Figure 1C,D). Consistently, in situ hybridization data showed strong *STAU2* mRNA signals in the cortex, hippocampus, and cerebellar Purkinje cell layer (Figure 1D). To directly examine endogenous STAU2 protein localization, we performed immunofluorescence staining on adult wild‐type mouse brain sections and found that STAU2 forms punctate structures in MAP2‐positive dendrites of cortical neurons, including layer III cortical neurons (Figure 1E). These results demonstrate that STAU2 is endogenously expressed in vivo and forms dendritic puncta in neurons.

### 2.2 STAU2 Forms Dynamic Granules to Deliver RNA in Neuronal Dendrites

**FIGURE 2:**
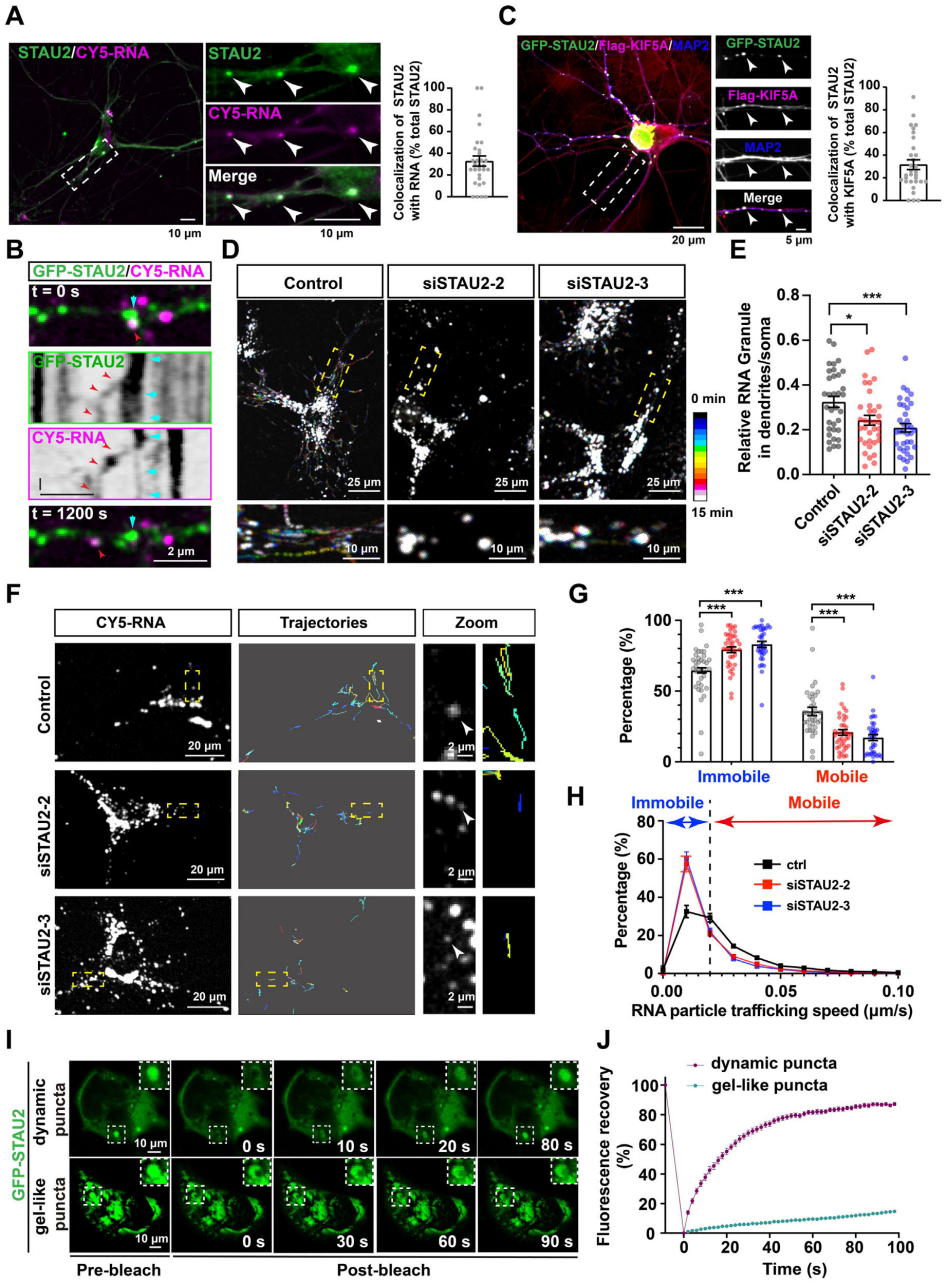
STAU2 forms RNP granules and facilitates RNA delivery in dendrites of primary neurons. (A) DIV9 rat hippocampal neurons were labeled with Cy5‐UTP to visualize total intracellular RNA (magenta). On DIV10, cells were fixed and immunostained for endogenous STAU2 (green). Left, RNA‐containing STAU2 puncta were observed in the soma and dendrites. The boxed region is magnified in the middle panels, highlighting dual‐positive RNA‐protein granules (STAU2^+^, green; RNA^+^, magenta), indicated by arrowheads. Scale bars: 10 µm. Right, quantification of STAU2–RNA colocalization in neuronal dendrites. Data are mean ± SEM (32.91 ± 4.66%; *n* = 30 neurons). (B) Representative kymographs of GFP‐STAU2 and Cy5‐labeled RNA in dendrites of DIV10 rat hippocampal neurons co‐expressing GFP‐STAU2 and CY5‐UTP. Red arrowheads indicate a moving GFP‐STAU2 and Cy5‐RNA dual‐positive granule, while blue arrows mark a stationary GFP‐STAU2 granule. Scale bars: x = 2 µm, y = 200 s. (C) Representative confocal images of DIV10 rat hippocampal neurons co‐expressing GFP‐STAU2 and Flag‐KIF5A. Left, three‐color images show GFP‐STAU2 (green), Flag‐KIF5A (magenta), and the dendritic marker MAP2 (blue) in neuronal dendrites; the boxed region is enlarged in the middle panels, with GFP‐STAU2 granules indicated by arrowheads. Scale bars, 20 µm (left) and 5 µm (middle). Right, quantification of STAU2–KIF5A colocalization in neuronal dendrites. Data are mean ± SEM (31.6 ± 4.23%; *n* = 31 neurons). (D) Live‐cell imaging of DIV10 rat hippocampal neurons expressing Cy5‐UTP and the indicated STAU2 siRNAs was performed using spinning disk confocal microscopy. Time‐lapse images of Cy5‐labeled RNA granules were acquired over 15 min. Representative projections illustrate RNA granule dynamics, with boxed dendritic regions amplified in the lower panels. RNA granules were color‐coded for different time frames. Scale bars: 25 µm (upper panels), 10 µm (lower panels). (E) Quantification of the dendrite‐to‐soma ratio of total RNA in (D). Data are presented as mean ± SEM (*n* = 34 [Control], 35 [siSTAU2‐2], and 36 [siSTAU2‐3]), ^*^ *p* < 0.05, ^***^ *p* < 0.001, Student's *t*‐test. (F) Representative images showing the mobility of individual Cy5‐labeled RNA granules in dendrites of DIV10 rat hippocampal neurons co‐expressing Cy5‐UTP and the indicated STAU2 siRNAs. The middle panels show RNA granule trajectories, with boxed regions magnified in the right panels. Scale bars, 20 µm. (G) Quantification of the percentage of mobile (>0.02 µm/s) and immobile (≤0.02 µm/s) Cy5‐labeled RNA granules in (F). Data are presented as mean ± SEM (*n* = 36 [Control]; *n* = 39 [siSTAU2‐2]; *n* = 35 [siSTAU2‐3]), ^***^ *p* < 0.001, Student's *t*‐test. (H) Distribution of the average speed of Cy5‐labeled RNA granules in (G), categorized as mobile (>0.02 µm/s) and immobile (≤0.02 µm/s). (I) Representative time‐lapse FRAP images of COS7 cells expressing GFP‐STAU2. The fluorescence signals of GFP‐STAU2 dynamic puncta were recovered in a short time after laser quenching, whereas gel‐like puncta showed nearly no fluorescence recovery. Scale bars: 10 µm. (J) FRAP recovery curves of GFP‐STAU2 dynamic puncta/gel‐like puncta of similar size from 30 cells after photobleaching. Timepoint 0 corresponds to the moment of the photobleaching laser pulse. Data are expressed as mean ± SEM.

To investigate how STAU2 mediates anterograde transport of target RNAs, we examined its endogenous distribution using specific antibodies in developing dendrites. In rat hippocampal neurons at DIV9, CY5‐UTP was transfected to label intracellular RNA [36]. On DIV10, STAU2 puncta co‐localized with CY5‐UTP‐labeled RNA were observed in both soma and dendrites of primary hippocampal neurons (Figure 2A). Importantly, exogenously expressed GFP‑tagged STAU2 forms punctate structures in developing dendrites, some of which colocalize and traffic with endogenous RNA in distal dendritic compartments (Figure 2B). We further found that GFP‐STAU2 could be robustly co‐immunoprecipitated by the Flag‐tagged C‐terminal cargo binding domain (aa 330–1027) of kinesin‐1 family member KIF5A (Figure S1A and Unprocessed Figure S1A), the major motor mediating plus end‐directed anterograde and long‐distance transport of membranous organelles or cargoes on neuronal microtubules [37, 38]. In line with this observation, Flag‐KIF5A exhibited well co‐localization with GFP‐STAU2 granules in the dendritic branches when co‐expressed in mature hippocampal neurons (Figure 2C), indicating that the STAU2/RNA granules could be recognized and delivered by KIF5A in dendrites of hippocampal neurons.

To further explore the role of STAU2, we employed two different siRNAs to knock down (KD) endogenous STAU2 (siSTAU2) in cultured neurons (Figure S1B and Unprocessed Figure S1B), and then measured the RNA trafficking efficiency by live‐imaging CY5‐labeled RNA granules in dendrites. We observed a significant reduction in anterograde trafficking events of CY5‐labeled RNA granules in dendrites of STAU2 KD neurons in both siSTAU2 groups compared to control neurons (Figure 2D). Consistently, the dendrite‐to‐soma ratio of CY5‐labeled RNA granules was significantly decreased in the STAU2 KD groups compared to the scrambled control group (Figure 2E). We also examined the dynamics of individual CY5‐labeled RNA granules by comparing the kinetics of automatically traced trajectories of these granules (Figure 2F). In dendrites of STAU2 KD neurons, the number of immobile RNA granules increased significantly, while the number of mobile RNA granules decreased (Figure 2F,G), impairing the dendritic delivery of RNA granules in neurons. This was reflected in the speed curves, with higher slow‐speed peaks (<0.02 µM/s) in the STAU2 KD groups compared to the control groups (Figure 2H). These data indicate STAU2 may play an important role in regulating the anterograde delivery of RNA in dendrites of developing neurons.

### 2.3 STAU2 Undergoes Phase Separation In Vitro and in Living Cells

To investigate the biophysical properties of those STAU2 granules, GFP‐STAU2 was expressed in COS7 cells at various levels, where the endogenous STAU2 level was extremely low [15]. As the amount of transfected GFP‐STAU2 plasmid increased or the expression time prolonged, GFP‐STAU2 formed many bright scattered puncta in the cytoplasm under the microscope (Figure S1C,D). Fluorescence recovery after the photobleaching (FRAP) analysis of these GFP‐STAU2 puncta showed that the fluorescence signal recovered in a short time (∼80% within ∼50 s; Figure 2I,J), indicating that GFP‐STAU2 in the condensed puncta were rapidly exchanging with surrounding proteins in the cytoplasm. Whereas high protein level induced the generation of gel‐like GFP‐STAU2 puncta in the cytoplasm (Figure S1C,D), and the GFP‐STAU2 signal in these puncta barely recovered in FRAP analysis (Figure 2I,J), suggesting a low dynamic, gel‐like state. These data indicate that GFP‐STAU2 could self‐organize to form enriched clusters in living cells in a concentration‐dependent manner, while even higher protein levels shift the STAU2 cluster from a dynamic liquid‐like state to a static gel‐like form.

**FIGURE 3:**
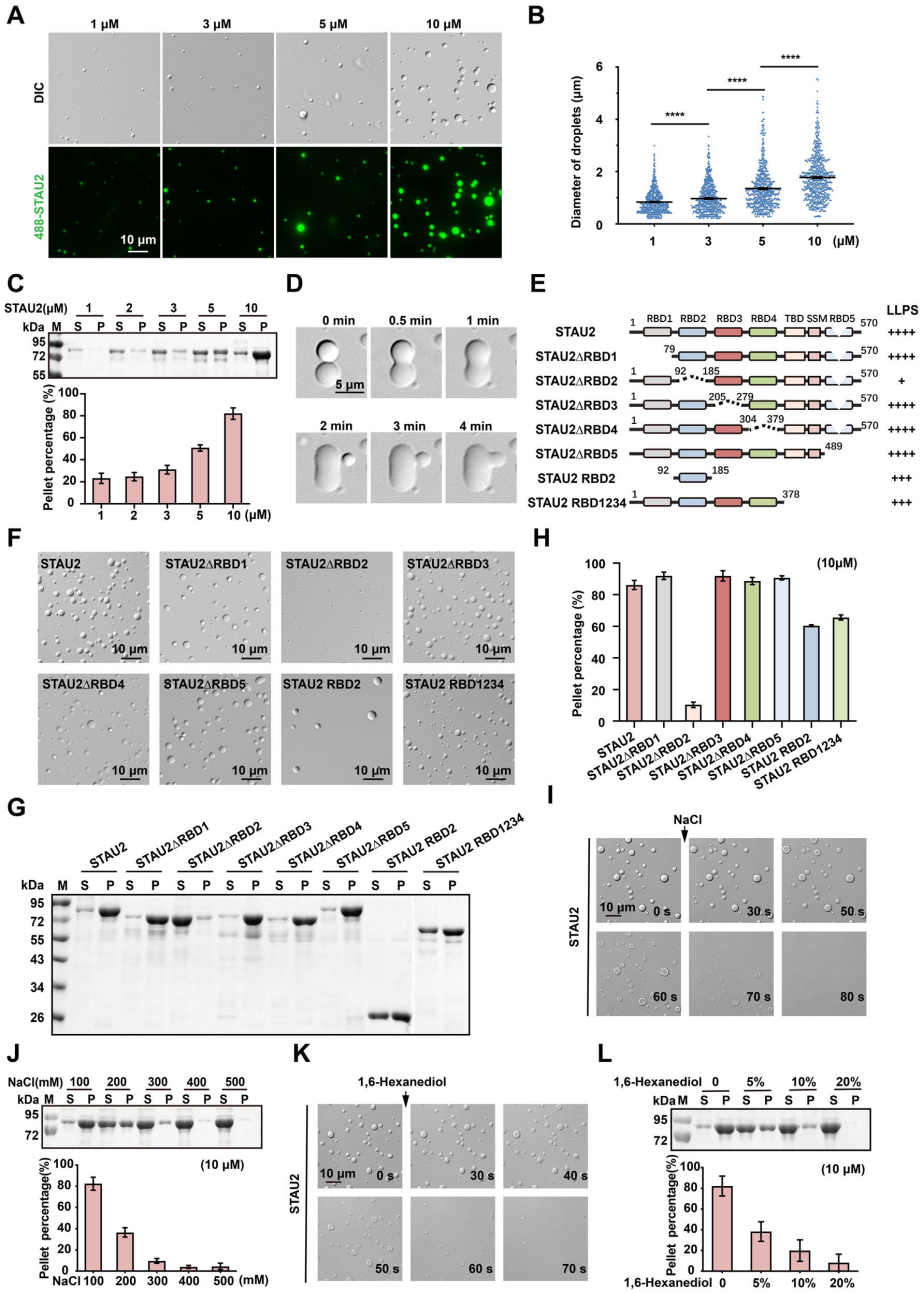
STAU2 undergoes liquid‐liquid phase separation in vitro. (A) DIC and fluorescence images of iFluor 488‐labeled STAU2. STAU2 undergoes LLPS in a protein concentration‐dependent manner. Scale bars: 10 µm. (B) Scatter plot representing the size of iFluor 488‐labeled STAU2 droplets at different protein concentrations. Data are shown as mean ± SEM, ^****^ *p* < 0.0001, One‐way ANOVA with Tukey's multiple comparison test. (C) SDS‐PAGE analysis and quantification of the distribution of STAU2 between the supernatant solution (S) and the liquid phase of the condensate (P) at different protein concentrations, assessed by sedimentation assay. Data are from three independent experiments and are expressed as mean ± SD. (D) STAU2 droplets fused into larger one over time under DIC microscopy. Scale bars: 5 µm. (E) Schematic illustration of various fragments of STAU2. RBD, RNA‐binding structural domain; TBD, Tubule‐binding domain; SSM, staufen‐swapping motifs. (F) Representative DIC images of various STAU2 fragments (10 µM). RBD2 mediates LLPS of STAU2. Scale bars: 10 µm. (G&H) SDS‐PAGE analysis (G) and quantification (H) of the distribution of various STAU2 fragments (10 µM) between the supernatant solution (S) and the condensed phase (P), assessed by sedimentation assay. Data are from three independent experiments and are expressed as mean ± SD. (I) Preformed STAU2 droplets (10 µM) rapidly dissolved upon the addition of 1 M NaCl buffer. Scale bars: 10 µm. (J) Sedimentation assay analysis of STAU2 at different salt concentrations. Data are from three independent experiments and are expressed as mean ± SD. (K) Preformed STAU2 droplets (10 µM) rapidly dissolved after the addition of 50% (w/v) 1,6‐hexanediol buffer. Scale bars: 10 µm. (L) Sedimentation assay analysis of STAU2 at different concentrations of 1,6‐hexanediol. Data are from three independent experiments and are expressed as mean ± SD.

Next, we purified the recombinant full‐length STAU2 protein from *Escherichia coli*. Differential interference contrast (DIC) microscopy and sedimentation assay analysis revealed that STAU2 spontaneously formed spherical condensed liquid droplets in a concentration‐dependent manner, as both the droplet sizes of iFluor 488‐labeld STAU2 and condensed phase fraction increased with increasing protein concentration (Figure 3A–C and Unprocessed Figure 3C). These STAU2 droplets exhibited a liquid‐like behavior and could fuse with each other to form a larger one (Figure 3D and Movie S1), which is a characteristic phenomenon of LLPS [39, 40, 41]. Thus, the LLPS property of STAU2 might be the mechanism of STAU2 granule formation.

### 2.4 A Conserved Insertion in RBD2 is Essential for STAU2 LLPS

STAU2 is a multi‐domain protein consisting of five tandem RBDs, a tubule‐binding domain (TBD), and a Staufen‐swapping motif (SSM) domain (Figure 3E). To find out the molecular mechanism of STAU2 LLPS, we constructed a series of STAU2 mutants by deleting each RBD, and tested their in vitro phase separation properties. DIC microscopy and sedimentation assay analysis revealed that deletion of RBD2 (STAU2 ΔRBD2) dramatically impaired STAU2 LLPS, deletion of both TBD‐SSM and RBD5 (STAU2 RBD1234) slightly weakened the droplet formation of STAU2, whereas deletion of either RBD1, RBD3, RBD4, or RBD5 had no observable influence on its phase separation property (Figure 3F–H and Unprocessed Figure 3G and Figure S2A and Unprocessed Figure S2A). It was further found that STAU2 RBD2 alone was sufficient to undergo spontaneous coacervation, and the pellet percentage in the condensed phase was comparable to that of STAU2 RBD1234 (∼60% at 10 µM protein concentration; Figure 3F–H and Unprocessed Figure 3G and Figure S2B and Unprocessed Figure S2B). Taken together, the data indicate that RBD2 is mainly responsible for the LLPS of STAU2.

**FIGURE 4:**
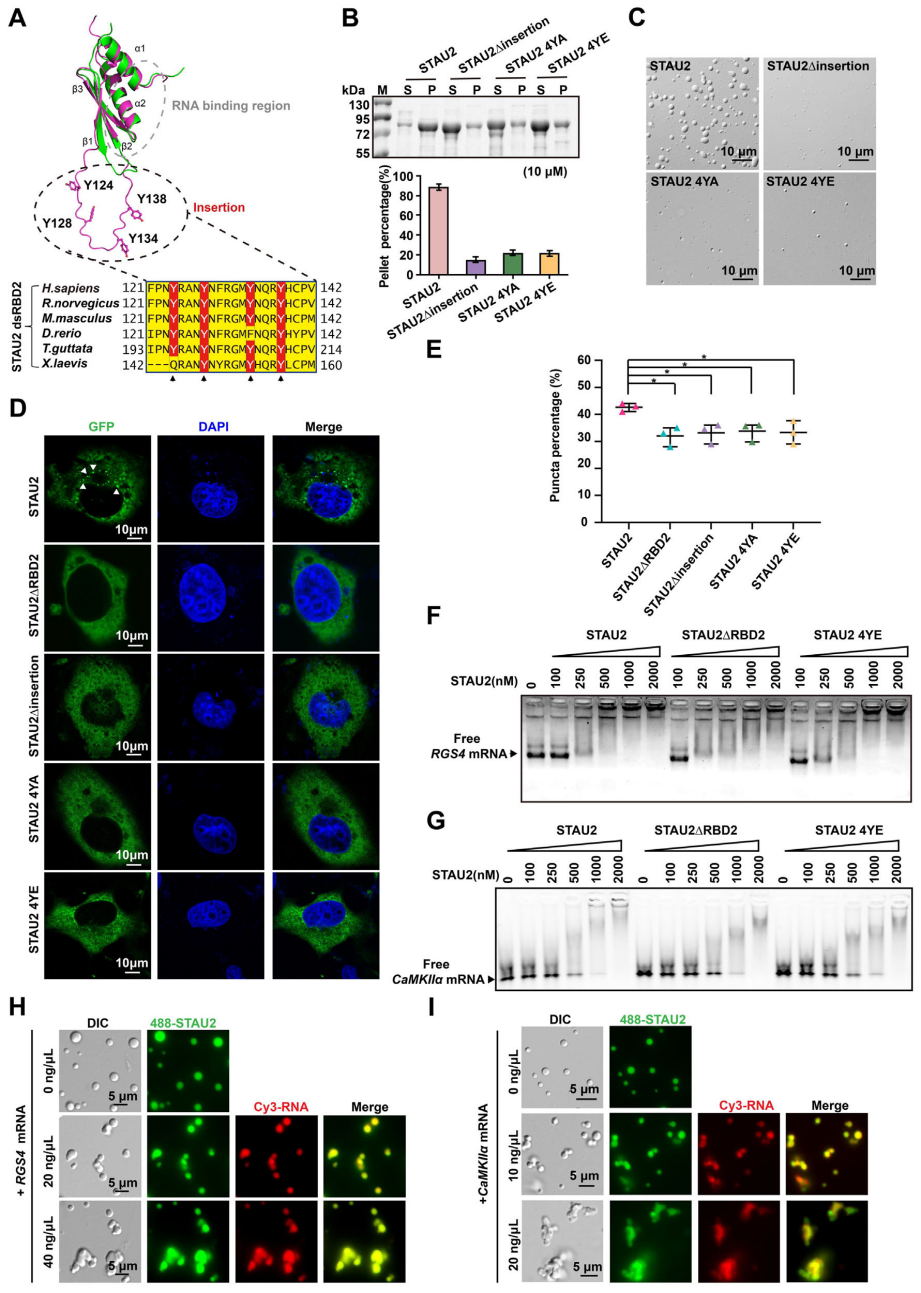
STAU2 binds to RNAs and forms gel‐like condensates. (A) Overlay of human STAU1 RBD3 (PDB ID: 6HTU, green) and human STAU2 RBD2 (AlphaFold predicted, magenta). The RBD2 insertion sequence is circled in a red dashed box, and the amino acid sequence is shown below. The key amino acids Y^124^Y^128^Y^134^Y^138^ are represented by a stick model (magenta), and the RNA‐binding region in RBD2 is indicated by a grey dashed oval. The bottom panel shows the sequence alignment of the insertion fragment in Stau RBD2 across various species. Conserved amino acids are highlighted in red and triangles below the sequence. (B) Sedimentation assay analysis of STAU2 or mutants (10 µM). Data are from three independent experiments and are expressed as mean ± SD. (C) Representative DIC images of STAU2 or mutants (10 µM). Scale bars: 10 µm. (D) Representative fluorescence images of COS7 cells expressing GFP‐STAU2 or mutants. White arrows indicate GFP‐STAU2 puncta. Scale bars: 10 µm. (E) Statistical data for puncta formation in (D). Data were obtained from three independent experimental cell culture batches, with 600 cells counted for each batch. Data are presented as mean ± SEM, ^*^ *p* < 0.05, one‐way ANOVA with Tukey's multiple comparison test. (E&F) EMSA experiments of STAU2, STAU2 ΔRBD2, or STAU2 4YE with *RGS4* 3'UTR mRNA (20 ng/µL) (E) or *CaMKIIα* mRNA (20 ng/µL) (F) in nondenaturing agarose gel at different protein concentrations. (H&I) DIC and fluorescence images of iFlour 488‐labeled STAU2 (10 µM) droplets in the presence of different concentrations of Cy3‐labeled *RGS4* 3'UTR mRNA (H) or *CaMKIIα* mRNA. Scale bars: 5 µm.

To understand the underlying mechanism of STAU2 LLPS, we treated STAU2 condensates with salt or 1,6‐hexanediol, two reagents widely used for interfering with protein phase separation driven by electrostatic or hydrophobic interactions, respectively [42, 43, 44]. The pre‐formed droplets of STAU2 or STAU2 RBD2 gradually collapsed and eventually dispersed into solution by addition of high‐concentration salt or 1,6‐hexanediol, respectively (Figure 3I–L and Unprocessed Figure 3J,L, Figure S2C and Movies S2 and S3), suggesting that electrostatic and hydrophobic interactions are the driven forces for the occurrence of STAU2 LLPS. Then we sought for the key residues located on RBD2 that mediated STAU2 LLPS. Compared to other classical RBD domains in Staufen family proteins (e.g., human STAU1 RBD3, PDB ID: 6HTU), there is an evolutionarily conserved insertion sequence between β1 and β2 of RBD2 (Figure 4A), which was suggested to interfere with the dsRNA binding ability of RBD2 in *Drosophila* Staufen [7]. We wondered whether this insertion was responsible for RBD2 LLPS. To test this hypothesis, we deleted this insertion sequence and found that LLPS ability of this STAU2 mutant (named as STAU2 Δinsertion) was dramatically weakened (Figure 4B,C), comparable to that of STAU2 ΔRBD2. Guided by the information that RBD2 LLPS was sensitive to 1,6‐hexanediol treatment, we substituted four highly conserved tyrosine residues Y^124^Y^128^Y^134^Y^138^ to alanine (referred to as STAU2 4YA) or glutamic acid (referred to as STAU2 4YE), and both mutations significantly impaired the droplet formation of both STAU2 RBD2 and STAU2 (Figure 4B,C and Unprocessed Figure 4B and Figure S2D,E and Unprocessed Figure S2D), indicating Tyr124, Tyr128, Tyr134, and Tyr138 from the insertion sequence are essential for STAU2 LLPS in vitro. Finally, we investigated the subcellular localization of the above STAU2 mutants in living COS7 cells. Consistent with in vitro phase separation results, all four mutants (GFP‐tagged STAU2 ΔRBD2, STAU2 Δinsertion, STAU2 4YA, and STAU2 4YE) exhibited a similar puncta formation property in COS7 cells, with a significant decrease of the puncta‐positive cell proportion compared to wild‐type STAU2 (Figure 4D,E).

Together, four tyrosine residues in a conserved insertion of RBD2 are essential for STAU2 to phase separate.

### 2.5 RNA Recruitment Causes Gelation of STAU2 Condensates

As a key regulator in neuronal RNA metabolism, STAU2 binds directly to diverse mRNAs via its RBD domains, including RBD2, which drives its LLPS [20]. Next, we explored whether the dsRNA binding property of STAU2 could be interfered with by its LLPS property. To test this hypothesis, we chose two STAU2 target mRNAs, *RGS4* mRNA and *CaMKIIα* mRNA, which are involved in dendritic spine development, neuronal signaling, and synaptic plasticity [23, 36, 45, 46]. Previous studies have shown that STAU2 interacts directly with the stem‐loop structure formed by the Stau‐recognized structures (SRSs) motif in the 3' untranslated region of the *RGS4* mRNA (*RGS4* 3'UTR) [20, 21, 27] or intron 16‐containing *CaMKIIα* mRNA [23], to stabilize these RNAs for dendritic transport. We generated *RGS4* 3'UTR mRNA (referred to as *RGS4* mRNA) and intron16‐containing *CaMKIIα* mRNA (referred to as *CaMKIIα* mRNA) by in vitro transcription. Consistent with the previous report [20], electrophoretic mobility shift assay (EMSA) confirmed that RBD2, RBD3, and RBD4 (but not RBD1 or RBD5) of STAU2 bound directly to *RGS4* mRNA in vitro (Figure S2F and Unprocessed Figure S2F). Nevertheless, the STAU2 ΔRBD2 mutant bound to *RGS4* or *CaMKIIα* mRNA equally well as the wild‐type STAU2, possibly due to the dominant binding of RBD34 towards target RNAs (Figure 4F,G and Unprocessed Figure 4F,G) [20]. Importantly, the insertion fragment of STAU2 RBD2 essential for its LLPS was outside its reported RNA binding sites (Figure 4A) [20]. Consistently, STAU2 4YE mutants showed a comparable binding to *RGS4* mRNA or *CaMKIIα* mRNA compared to STAU2 (Figure 4F,G and Unprocessed Figure 4F,G). These data indicate that the two LLPS‐deficient mutations did not significantly interfere with the RNA‐binding property of STAU2.

**FIGURE 5:**
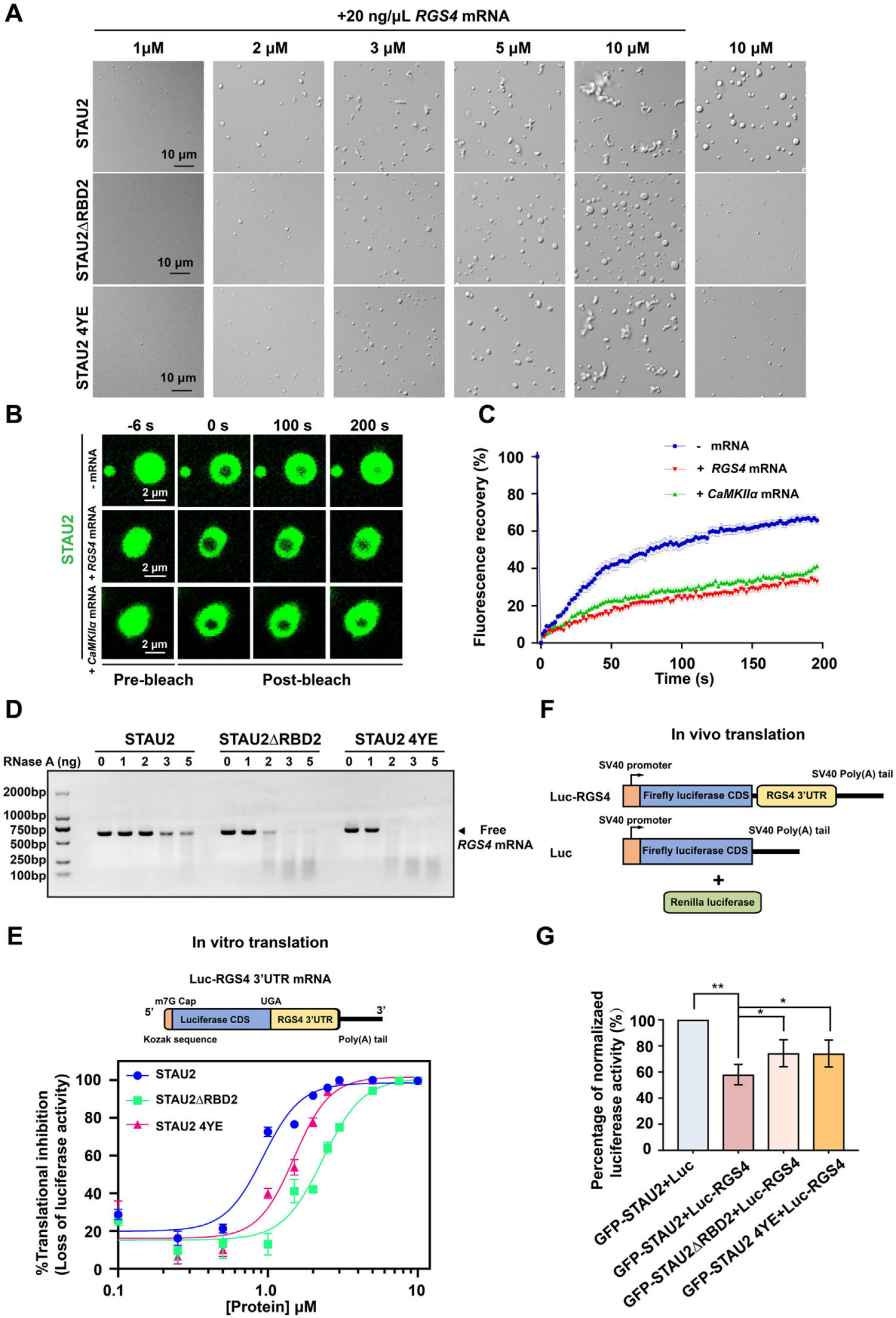
STAU2 binds to RNAs and is involved in translation inhibition. (A) Representative DIC images of STAU2, STAU2 ΔRBD2, or STAU2 4YE with *RGS4* 3'UTR mRNA (20 ng/µL) at different protein concentrations. Scale bars: 10 µm. (B) Representative time‐lapse in vitro FRAP images of iFlour 488‐labeled STAU2 (10 µM) droplets in the absence or presence of *RGS4* 3'UTR mRNA (20 ng/µL) or *CaMKIIα* mRNA (20 ng/µL). Scale bars: 2 µm. (C) FRAP recovery curves of 30 STAU2 droplets of similar size in the absence or presence of *RGS4* 3'UTR mRNA (20 ng/µL) or *CaMKIIα* mRNA (20 ng/µL) after photobleaching. Data are expressed as mean ± SEM. (D) In vitro RNA stability assay of *RGS4* mRNA (20 ng/µL) in the presence of STAU2 or its mutants (10 µM). STAU2 condensate recruits and protects target mRNA from degradation. (E) In vitro translation assay of STAU2 and its mutants using luciferase*‐RGS4* 3'UTR mRNA. A schematic diagram of luciferase*‐RGS4* 3'UTR mRNA in vitro transcription is shown at the top. The bottom panel illustrates the translation levels of Luc‐RGS4 3'UTR in the presence of STAU2 or its mutants. Data are from three independent experiments and are expressed as mean ± SD. (F) Schematic diagram of the dual‐luciferase reporter plasmid. Firefly luciferase reporter plasmid containing *RGS4* 3'UTR sequence referred to as Luc‐RGS4, control Firefly luciferase reporter plasmid, referred to as Luc. (G) Dual‐luciferase reporter assay to evaluate the effect of GFP‐STAU2 and its mutants on the translation of a reporter plasmid containing the *RGS4* 3'UTR sequence. Data are derived from four independent experiments and are expressed as mean ± SD, ^*^ *p* < 0.05, ^**^ *p* < 0.01, one‐way ANOVA, and Tukey's multiple comparison test.

Based on previous studies [20, 47, 48] and combined sequence and structural alignment analyses, we generated a series of RNA‐binding‑deficient STAU2 mutants, including RBD1 F40A (RBD1 mut), RBD2 F157, H169A (RBD2 mut), RBD3 H235, K237, K257, K258, K261A (RBD3 mut), RBD4 R336, R338, K358, K359, K362A (RBD4 mut), and a combined full‑length STAU2 mutant harboring all the above RNA‑binding disruptive substitutions (STAU2 mut). These mutants largely lost the ability to bind *RGS4* mRNA (Figure S2F and Unprocessed Figure S2F). Moreover, sedimentation assays confirmed that STAU2 mut still retained robust phase‐separation capacity at 10 µM concentration (Figure S2G and Unprocessed Figure S2G). However, when expressed in neurons, GFP‐STAU2 mut exhibited markedly reduced formation of dendritic puncta and remained largely diffuse throughout dendrites (Figure 7B,C), likely because impaired RNA binding prevents local STAU2 enrichment from reaching the threshold required for robust condensate assembly. In sharp contrast, GFP‐STAU2 WT formed prominent RNA granules at comparable expression levels. We reason that this difference arises because RNA‐binding significantly promotes STAU2 phase separation (Figure 5A and Figure S2H and Unprocessed Figure S2H). As a result, in neurons, this mutant appears to be compromised not only in RNA binding but also in dendritic condensate formation, displaying a phenotype similar to that of the GFP control (Figure 7 and 8). These results suggest that, in dendrites, STAU2 condensation and target mRNA binding are functionally coupled, and both contribute to efficient target mRNA localization.

When incubating iFluor 488‐labeled STAU2 with Cy3‐labelled *RGS4* mRNA or *CaMKIIα* mRNA, the target mRNA could be effectively recruited and enriched into the STAU2 condensates (Figure 4H,I). We noticed that the STAU2 condensate exhibited a liquid‐to‐gel/aggregate transition upon the addition of an increasing amount of target mRNAs, and the situation worsened as the STAU2 concentration increased (Figures 4H,I and 5A, and Figure S2H). FRAP analysis also demonstrated that the fluorescence signal of iFluor 488‐STAU2 droplets could rapidly recover after photobleaching, whereas the recovery rate of STAU2 fluorescence signal was dramatically slowed down in the presence of the *RGS4* mRNA or *CaMKIIα* mRNA (Figure 5B,C), indicating that the RNA recruitment changes the internal dynamics of STAU2 condensates. Intriguingly, the presence of target mRNA significantly enhanced the phase separation capability of both STAU2 ΔRBD2 and STAU2 4YE mutants, and the two mutants exhibited alleviated liquid‐to‐gel/aggregate transition induced by target mRNA (Figure 5A), implying a regulatory role of RNA binding in the phase separation/phase transition of STAU2.

### 2.6 STAU2 Condensates Stabilize Embedded mRNA and Inhibit its Translation

Alterations in the physical properties of condensates often involve functional changes. For instance, the liquid‐to‐gel/solid transition of FUS, hnRNPA1, Whi3, and other IDR‐containing proteins is correlated with neurodegenerative diseases [40, 49, 50, 51, 52, 53]. We investigated whether the liquid‐to‐gel/aggregate transition of STAU2, induced by target RNAs, plays a role in regulating mRNA metabolism using *RGS4* mRNA as a model. First, we confirmed that STAU2 binding enhances the stability of *RGS4* mRNA [20, 21, 27], and this effect is at least partially dependent on the formation of STAU2 condensate (Figure 5D and Unprocessed Figure 5D). Then we performed an in vitro rabbit reticulocyte lysate translation assay [49] in the presence of various concentrations of STAU2 or the LLPS‐deficient mutants, and luminescence was monitored as an indicator for luciferase*‐RGS4* 3'UTR mRNA translation (Figure 5E). The translation level of luciferase*‐RGS4* 3'UTR mRNA suddenly decreased when STAU2 reached its critical concentration (∼1 µM) for LLPS in the translation system (IC50 ∼0.9 µM, Hillscope = 3.437; Figure 5A,E). Whereas the LLPS‐deficient STAU2 ΔRBD2 and STAU2 4YE mutants exhibited significantly impaired inhibition effects on RNA translation (with IC50 ∼2.3 µM and 1.5 µM, Hillscope = 3.241 and 3.629, respectively). Noted that in comparison with STAU2 ΔRBD2 and STAU2 4YE, STAU2 showed much more severe target RNA‐induced gelation/aggregation at the same protein concentration, pointing to a positive correlation of STAU2 phase transition with its ability to inhibit target mRNA translation (Figure 5A,E).

Next, we performed a dual‐luciferase reporter assay in vivo to further verify the potential role of STAU2 condensates in regulating *RGS4* mRNA translation. We constructed the Firefly luciferase reporter plasmid with or without the *RGS4* 3'UTR sequence (referred to as Luc or Luc‐RGS4, respectively) and co‐transfected with Renilla luciferase plasmid (as internal control) and GFP‐tagged STAU2, STAU2 ΔRBD2, or STAU2 4YE in HEK293T cells, respectively (Figure 5F and Figure S2I and Unprocessed Figure S2I). The results showed that co‐transfection with GFP‐STAU2 and Luc‐RGS4 resulted in significantly lower Firefly luciferase activity than that of Luc, suggesting that STAU2 inhibited the translation of Firefly luciferase by binding to the *RGS4* 3'UTR (Figure 5G). Moreover, STAU2‐mediated translation inhibition effect could be significantly weakened when Luc‐RGS4 was co‐expressed with the LLPS‐deficient STAU2 ΔRBD2 or STAU2 4YE mutants.

### 2.7 Disturbance of STAU2 Condensation Affects its Capacity to Assemble RGS4 or CaMKII RNA Granules in Dendrites

**FIGURE 6:**
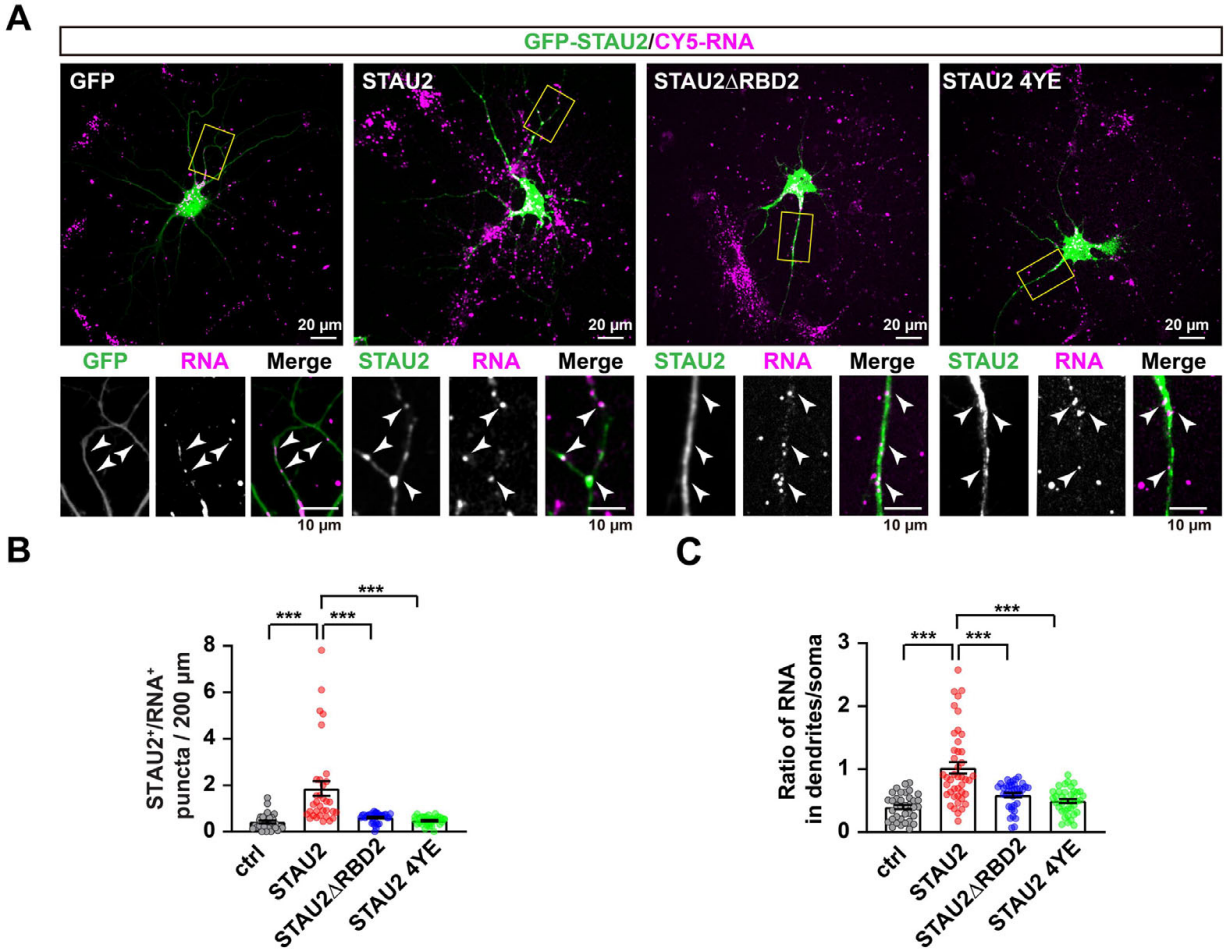
Phase separation‐related mutants of STAU2 impair its capacity to deliver RNA in dendrites of hippocampal neurons. (A) Live imaging of DIV7 rat hippocampal neurons co‐transfected with CY5‐UTP and GFP, GFP‐STAU2, GFP‐STAU2 ΔRBD2, or GFP‐STAU2 4YE. Representative dual‐color time‐lapse confocal images show the localization and movement of CY5‐UTP‐labeled RNA granules (magenta) in dendrites of neurons expressing control or STAU2 constructs (green). Boxed dendritic regions are amplified in the bottom panels, with RNA granules indicated by arrowheads. Scale bars: 20 µm (upper panels) and 10 µm (lower panels). (B) Quantification of the relative number of dual‐positive puncta (STAU2^+^/RNA^+^) in (A). Data are presented as mean ± SEM (*n* = 32 [Control], *n* = 33 [STAU2], *n* = 32 [STAU2 ΔRBD2], *n* = 33 [STAU2 4YE]), ^***^ *p* < 0.001, Student's *t*‐test. (C) Quantification of the dendrite‐to‐soma ratio of total RNA granules in A. Data are presented as mean ± SEM (*n* = 37 [Control], *n* = 44 [STAU2], *n* = 37 [STAU2 ΔRBD2], *n* = 39 [STAU2 4YE]), ^***^ *p* < 0.001, Student's *t*‐test.

We next investigated whether STAU2‐mediated RNP assembly and transport in neuronal dendrites are dependent on its LLPS property. Firstly, in DIV 6 hippocampal neurons, we examined the RNA correlation and the dendritic distribution of GFP‐STAU2 and two LLPS‐deficient mutants (GFP‐STAU2 ΔRBD2 and GFP‐STAU2 4YE) in the proximal 200 µm of dendrites. In live DIV6 hippocampal neurons expressing control GFP or GFP‐tagged STAU2 proteins, only wild‐type STAU2 existed in granules, whereas STAU2 ΔRBD2 or STAU2 4YE mutants were more diffusely distributed throughout the dendrites (Figure 6A). Furthermore, STAU2 granules exhibited significant overlap with CY5‐labeled RNA granules, whereas the two LLPS‐deficient STAU2 mutants did not (Figure 6B). Interestingly, we noted that overexpression of wild‐type STAU2 (but not the two LLPS‐deficient STAU2 mutants) enhanced the dendritic distribution of CY5‐labeled RNA in neurons (Figure 6C), indicating that defects in the LLPS capacity of STAU2 may affect its RNA cargo delivery in dendrites.

We noticed that the granule formation of STAU2 in COS7 cells (Figure 4E) showed a much smaller decrease with the variants than that observed in the in vitro phase separation assays (Figures 3H and 4B), whereas puncta formation was profoundly disrupted in neuronal dendrites (Figure 6B). Given that both self‐oligomerization and RNA‐binding contribute to STAU2 condensation (Figure 5A), we propose that the discrepancy among the robust in vitro phase‐separation capacity, the mild phenotype in COS7 cells, and the severe defect in neuronal dendrites may reflect differences in the local availability of STAU2 target RNAs. Specifically, COS7 cells and the neuronal soma may provide a relatively RNA‐rich environment that partially supports condensate formation even when RNA binding is weakened, whereas dendrites contain much lower levels of STAU2 target mRNAs [21, 54, 55].

**FIGURE 7:**
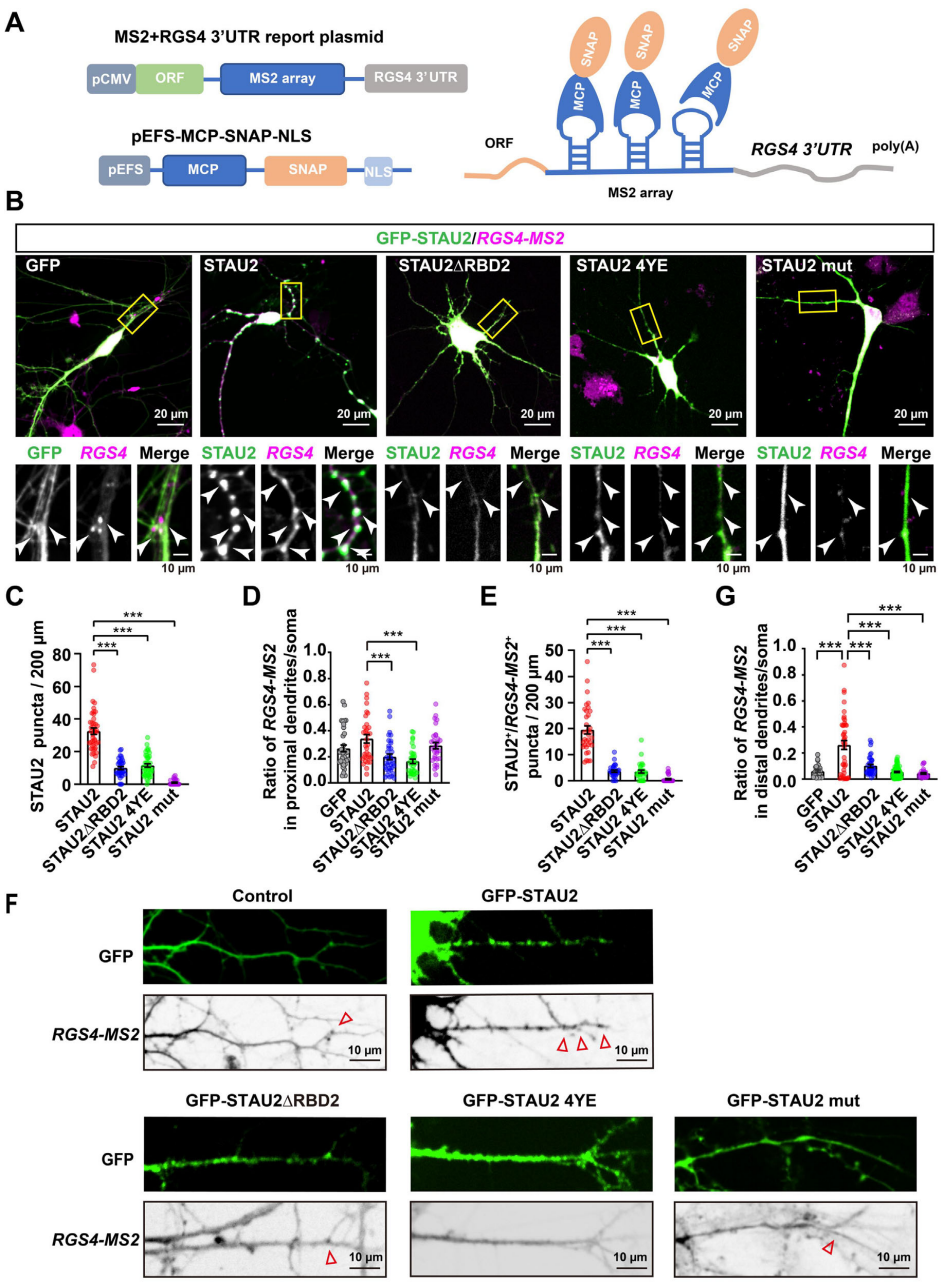
STAU2 granules colocalize with *RGS4* mRNA in dendrites of cultured hippocampal neurons. (A) Scheme of the *RGS4‐MS2* and MCP‐SNAP expression cassettes (left) and the MS2 mRNA reporter system (right). pEFS (promoter), ORF (open reading frame), NLS (nuclear localization signal), MCP (MS2 coat protein), UTR (untranslated region). (B) *RGS4‐MS2* and MCP‐SNAP mRNA reporter systems were co‐transfected with GFP, GFP‐STAU2, GFP‐STAU2 ΔRBD2, GFP‐STAU2 4YE, or GFP‐STAU2 mut in DIV9 rat hippocampal neurons. Live‐cell imaging was performed on DIV10 using spinning disc confocal microscopy. Representative dual‐color time‐lapse images show the subcellular localization and movement of STAU2 (green) and *RGS4‐MS2* (magenta) puncta in dendrites of neurons expressing the indicated constructs. Boxed regions are magnified in the bottom panels, highlighting *RGS4‐MS2*‐positive puncta (*RGS4* mRNA), indicated by arrowheads. Scale bars: 20 µm (upper panels), 10 µm (lower panels). (C) Quantification of STAU2 puncta density in (B). Data are presented as mean ± SEM (*n* = 43 [STAU2], *n* = 34 [STAU2 ΔRBD2], *n* = 41 [STAU2 4YE], *n* = 30 [STAU2 mut]), ^***^ *p* < 0.001, Student's *t*‐test. (D) Quantification of the proximal dendrite‐to‐soma ratio of *RGS4‐MS2* intensity in B. Data are presented as mean ± SEM (*n* = 35 [Control], *n* = 33 [STAU2], *n* = 36 [STAU2 ΔRBD2], *n* = 35 [STAU2 4YE], *n* = 30 [STAU2 mut]), ^***^ *p* < 0.001, Student's *t*‐test. (E) Quantification of the relative amount of dual‐positive (STAU2^+^/RGS4^+^) puncta in (B). Data are presented as mean ± SEM (*n* = 34 [STAU2], *n* = 32 [STAU2 ΔRBD2], *n* = 32 [STAU2 4YE], *n* = 30 [STAU2 mut]), ^***^ *p* < 0.001, Student's *t*‐test. (F) On DIV9, rat hippocampal neurons were co‐transfected with *RGS4‐MS2* and MCP‐SNAP mRNA reporter systems, along with GFP, GFP‐STAU2, GFP‐STAU2 ΔRBD2, GFP‐STAU2 4YE, or GFP‐STAU2 mut constructs. Live‐cell imaging of GFP and *RGS4‐MS2* was performed on DIV10 using spinning disc confocal microscopy. Representative images show the distribution of GFP‐STAU2 (upper panels) and *RGS4‐MS2* (lower panels) in dendrites of hippocampal neurons. Red triangles indicate *RGS4‐MS2* granules in distal dendrites. Scale bars: 10 µm. (G) Quantification of the distal dendrite‐to‐soma ratio of *RGS4‐MS2* intensity in (F). Data are presented as mean ± SEM (*n* = 31 [GFP], *n* = 43 [STAU2], *n* = 38 [STAU2 ΔRBD2], *n* = 72 [STAU2 4YE], *n* = 30 [STAU2 mut]), ^***^ *p* < 0.001, Student's *t*‐test.

To examine the efficiency of the above STAU2 proteins in delivering specific functional mRNA cargoes in proximal dendrites of developing neurons, we employed an MS2‐MCP‐based mRNA reporting system [21] to live‐trace the trafficking of *RGS4* and *CaMKII* mRNAs (Figure 7A and Figure S3A). Wild‐type STAU2 exhibited a higher granule formation capacity than the two LLPS‐deficient STAU2 mutants (STAU2 ΔRBD2 and STAU2 4YE) (Figure 7B,C and Figure S3B,C). Notably, *RGS4‐MS2*, representing the *RGS4* mRNA, formed significantly more dendritic granules in wild‐type STAU2 overexpressing neurons than in STAU2 ΔRBD2 or STAU2 4YE‐expressing neurons (Figure 7D), indicating that wild‐type STAU2 has a superior capacity to assemble and deliver *RGS4* mRNA granules in proximal dendrites. As expected, more STAU2 granules containing *RGS4‐MS2* were observed in wild‐type STAU2 overexpressing neurons than in the two STAU2 mutant expressing neurons (Figure 7E). Similar results were observed for *CaMKII* mRNA, as detected using the *CaMKII‐MS2* reporter (Figure S3A). It formed more granules in dendrites and showed higher colocalization with wild‐type STAU2 than the two LLPS‐deficient mutants STAU2 ΔRBD2 or STAU2 4YE (Figure 5D,E and Figure S3B).

Next, we compared the density of *RGS4‐MS2* and *CaMKII‐MS2* granules in the distal dendrites of wild‐type STAU2, STAU2 ΔRBD2, or STAU2 4YE‐expressing neurons on DIV 10 (Figure 7F). In the most distal 100 µm of dendrites, we found that the wild‐type STAU2 granules were significantly denser than those of the mutants (Figure 7F). Similar to STAU2, there were also more *RGS4‐MS2* granules existing in distal dendrites in wild‐type STAU2‐expressing neurons than in STAU2 ΔRBD2 or STAU2 4YE‐expressing neurons (Figure 7G), indicating that wild‐type STAU2 promotes the distal delivery of *RGS4* mRNA. Regarding *CaMKII* mRNA, different from *RGS4*, the overexpression of all STAU2 constructs, including wild‐type, ΔRBD2, and 4YE mutants, resulted in a significant enhancement in the density of *CaMKII‐MS2* in distal dendrites compared to GFP vector‐expressing control neurons (Figure S3G,H). These data suggest that STAU2 may prioritize the delivery of different mRNA cargoes to distal dendrites with varying efficiency.

Taken together, the above results suggest that the assembly and delivery of certain STAU2 target mRNAs occur in an LLPS‐dependent manner in neuronal dendrites.

### 2.8 Smaller STAU2/RNA Granules Exhibit Higher Mobility and Dynamics

**FIGURE 8:**
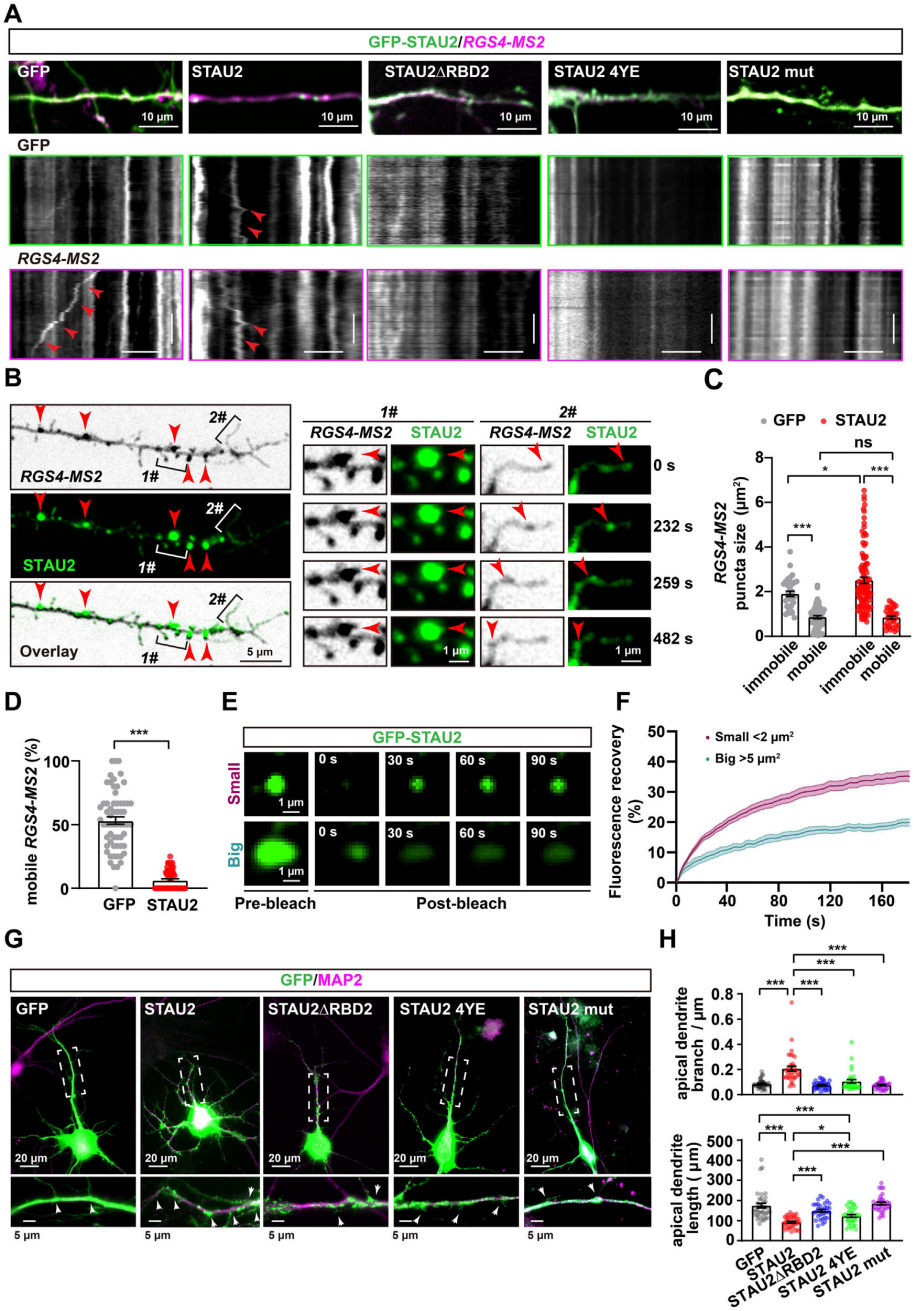
Ectopically expressed STAU2 regulates the dendritic trafficking of its mRNA cargo *RGS4* in cultured hippocampal neurons. (A) Representative kymographs of *RGS4‐MS2* granules in the dendrites of DIV10 rat hippocampal neurons transfected with the indicated constructs, with mobile *RGS4‐MS2* granules indicated by red arrowheads. Scale bars: x = 10 µm, y = 5 min. (B) Representative images illustrating the colocalization and co‐trafficking of STAU2 with *RGS4‐MS2* mRNA in RNP granules in distal dendrites of GFP‐STAU2 expressing DIV10 rat hippocampal neurons, with arrowheads indicating their colocalizations. Time‐lapse images of the bracketed region are magnified in the right panels. Region #1 shows a stationary STAU2 punctum colocalized with an *RGS4‐MS2* mRNA granule. Region #2 shows an *RGS4‐MS2* mRNA granule co‐trafficking with STAU2. Scale bars: 5 µm (left), 1 µm (right). (C) Quantification of the *RGS4‐MS2* puncta size from (B). Data are presented as mean ± SEM (*n* = 32 [immobile, Control], *n* = 52 [mobile, Control], *n* = 102 [immobile, STAU2], *n* = 32 [mobile, STAU2]. ^***^ *p* < 0.001, Student's *t*‐test). (D) Quantification of the percentage of mobile *RGS4‐MS2* puncta among 3–14 granules per dendrite in control or GFP‐STAU2 overexpressing neurons, as shown in (B). Data are presented as mean ± SEM; (*n* = 61 dendrites from 32 neurons for Control, *n* = 64 dendrites from 32 neurons for STAU2. ^*^ *p* < 0.001, Student's *t*‐test). (E) Representative time‐lapse images illustrating the FRAP rates in different‐sized GFP‐STAU2 granules. The boxed regions represent either large (upper) or small (lower) granules, which are amplified in the bottom panels. Scale bars: 1 µm. (F) FRAP rates of “big” and “small” granules. The purple curve (“small”, area < 2 µm^2^) and cyan curve (“big”, area > 5 µm^2^) represent the average FRAP rates for the two groups of STAU2 granules. Data are shown as mean ± SEM (“Small”, *n* = 67 ROIs from 22 neurons; “Big”, *n* = 27 ROIs from 17 neurons. ^***^ *p* < 0.001, Student's *t*‐test). (G) Representative confocal images of apical dendrites from DIV10 rat hippocampal neurons expressing GFP, GFP‐STAU2, GFP‐STAU2 ΔRBD2, GFP‐STAU2 4YE, or GFP‐STAU2 mut. Boxed regions are magnified in the bottom panels, with STAU2 granules indicated by arrowheads. MAP2 (magenta) was used as a dendritic marker. Scale bars: 20 µm. **(H)** Quantification of branch density (upper panel) and length (lower panel) of the apical dendrites in STAU2 and mutant overexpressing neurons shown in (**G)**. Data are shown as mean ± SEM (Branch, *n* = 38 [GFP], *n* = 36 [STAU2], *n* = 32 [STAU2 ΔRBD2], *n* = 33 [STAU2 4YE], and *n* = 34 [STAU2 mut], respectively; Length, *n* = 36 [GFP], *n* = 39 [STAU2], *n* = 32 [STAU2 ΔRBD2], *n* = 33 [STAU2 4YE], and *n* = 33 [STAU2 mut], respectively), ^**^ *p* < 0.01, ^***^ *p* < 0.001, Student's *t*‐test.

Interestingly, in dendrites of neurons expressing wild‐type GFP‐STAU2, the colocalized STAU2 and *RGS4‐MS2* tend to form large, immobile puncta, particularly in protruding and branching regions (Figures 7B and 8A,B), however, only the smaller puncta remain mobile, demonstrating the co‐trafficking of STAU2 and *RGS4‐MS2* (Figure 8B). Similar observations were made for STAU2/*CaMKII* granules (Figure S3F). In contrast, in neurons co‐transfected with control GFP and *RGS4‐MS2*, a larger portion of *RGS4‐MS2* granules were small and mobile (Figures 7B and 8A–D). The results suggest that the functional capacity of STAU2 granules in mRNA delivery may be modulated by their size and physical properties, which are in turn determined by the specific composition and concentration of their molecular components.

To test the above hypothesis, we first compared the dynamics of GFP‐STAU2 granules of various sizes in cultured hippocampal neurons. We observed a negative correlation of the FRAP recovery rate of individual STAU2 granules relative to their size (area), indicating that STAU2 molecules in smaller granules exhibit higher dynamics. Granules smaller than 2 µm^2^ were classified as “small”, while those larger than 5 µm^2^ were designated as “big”. Comparing their FRAP rates revealed that small granules recovered significantly faster than their larger counterparts (Figure 8E‐F). These distinct dynamic properties of STAU2 granules with different sizes seem to correlate with their transport efficiency in the dendrites of hippocampal neurons (Figure 8B–D).

In conclusion, the interplay between STAU2 and targets mRNAs along with their local concentrations regulates the assembly and physical property of STAU2/RNA granules, which may ultimately determine their efficiencies in dendritic trafficking.

### 2.9 STAU2 Condensation Status Affects the Dendritic Growth in Hippocampal Neurons

The contradictory observations that the density of *RGS4‐MS2* was much higher in distal dendrites of neurons overexpressing STAU2 (Figure 7F,G), and large STAU2/*RGS4‐MS2* granules were prone to be less mobile for distal transport (Figure 8A–D), prompted us to investigate the physiological relevance of STAU2 condensation in regulating dendritic development in hippocampal neurons. We examined dendritic growth in neurons expressing GFP control, GFP‐STAU2, or the LLPS‐deficient mutants STAU2 ΔRBD2 or STAU2 4YE. Our findings revealed that neurons expressing STAU2 had significantly shorter but more branched dendrites compared to control neurons. In contrast, over‐expression of the LLPS‐deficient mutants of STAU2, especially ΔRBD2, had no significant effect on dendritic morphology (Figure 8G,H). These data suggest that while STAU2 overexpression‐induced condensation may promote the assembly of RNA granules, the long‐distance delivery efficiency of these STAU2‐RNPs along developing dendrites is impaired by their size, which is positively correlated with STAU2 levels. The inefficient distal delivery of STAU2‐RNPs could promote dendritic branching at the expense of hindering dendritic elongation.

### 2.10 Activity‐Dependent STAU2 Condensation Controls Synaptic mRNA Translation

Previous studies showed that STAU2 associates with neuronal mRNA granules containing stalled ribosomes and local translation could be initiated upon synaptic signaling [56, 57]. Our colocalization analysis using the ribosomal marker RPS6 indicates that a substantial fraction of STAU2/RGS4 granules is ribosome‐associated, supporting the idea that STAU2 condensates overlap with ribosome‐containing neuronal RNP assemblies (Figure S4A).

**FIGURE 9:**
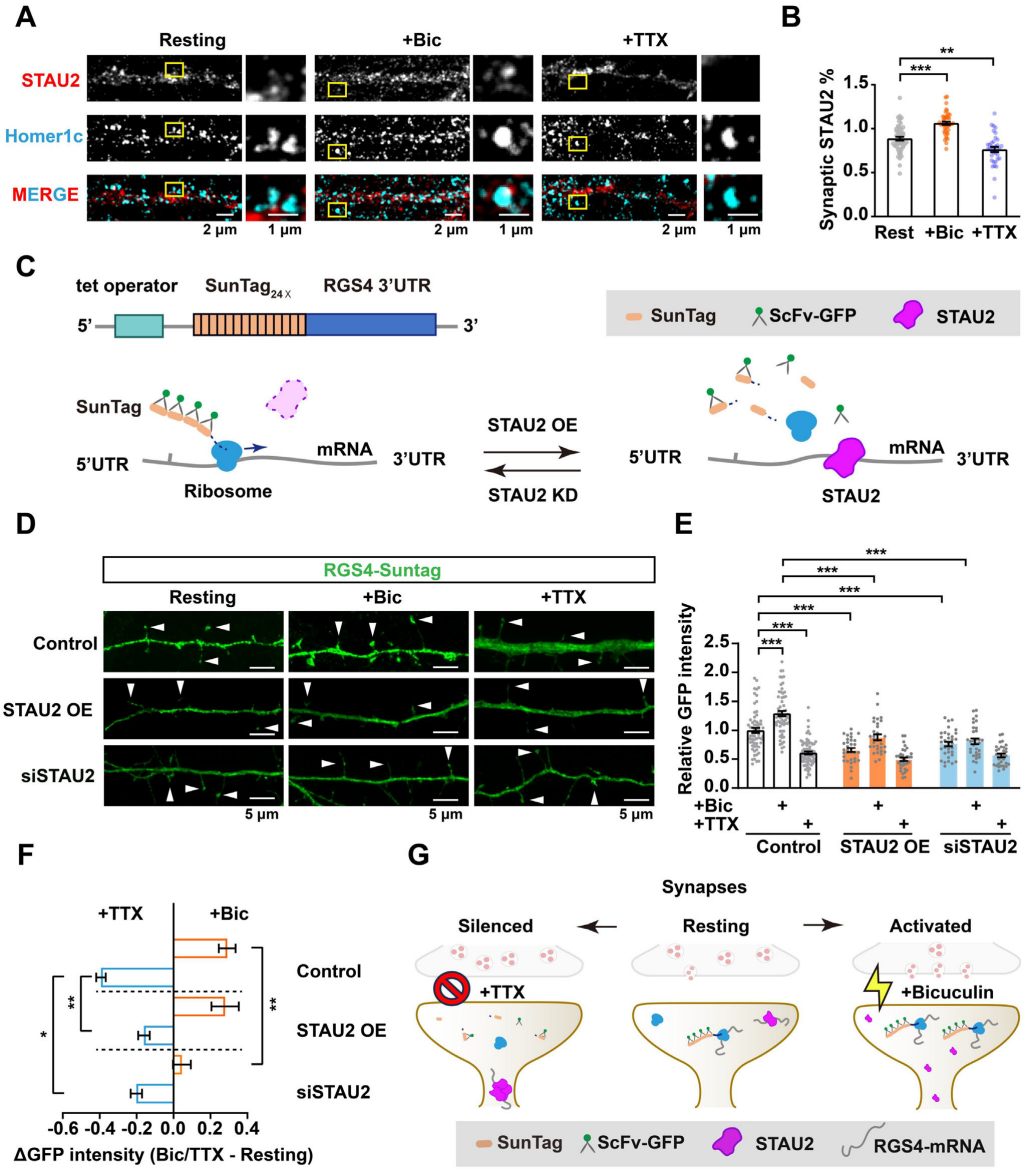
Synaptic activity controls local translation of *RGS4* mRNA in a STAU2‐dependent manner. (A) Representative confocal images of DIV14 rat hippocampal neurons showing endogenous STAU2 (red) and the postsynaptic marker Homer1c (cyan) under three conditions: untreated (“resting”), excitatory (“+Bic”), or silenced (“+TTX”). Scale bars, 2 µm (left) and 1 µm (right). (B) Quantification of the percentage of endogenous STAU2 localized to dendritic spines. Data are mean ± SEM (Rest, *n* = 50; +Bic, *n* = 48; +TTX, *n* = 36), ^**^ *p* < 0.01, ^***^ *p* < 0.001, one‐way ANOVA. (C) Schematic of the RGS4 SunTag reporter. Nascent RGS4 polypeptides carry tandem SunTag epitopes recognized by co‐expressed ScFv‐GFP (top). STAU2 overexpression (STAU2‐OE) or knockdown (siSTAU2) modulates the translation complex (bottom). (D) Representative confocal images of dendrites from DIV14 rat hippocampal neurons co‐expressing the SunTag reporter with either empty vector (Control), mCherry‐STAU2 (STAU2‐OE), or siRNA against STAU2 (siSTAU2) under the three synaptic conditions described above. Newly synthesized RGS4 peptides detected by ScFv‐GFP in dendritic spines are indicated by arrowheads. Scale bars, 5 µm. (E) Quantification of relative SunTag/ScFv‐GFP intensity in dendritic spines under the conditions shown in D. Values are normalized to the resting Control group. Data are mean ± SEM (*n* = 65, 64, 72, 33, 32, 34, 34, 32, 32 from left to right), ^***^ *p* < 0.001, one‐way ANOVA. (F) Fold change in nascent RGS4 synthesis in dendritic spines induced by excitatory (“+Bic”) or silencing (“+TTX”) treatments relative to resting conditions in Control, STAU2‐OE, and siSTAU2 neurons. Data are mean ± SEM (same n values as in E), ^*^ *p* < 0.05, ^**^ *p* < 0.01, one‐way ANOVA. (G) Model summarizing how synaptic activity bidirectionally regulates STAU2–RNP assembly and local RGS4 translation at synapses. Under silenced conditions (“+TTX”, left), STAU2 forms large condensates at postsynaptic sites and strongly represses RGS4 translation. At rest (middle), STAU2 forms baseline condensates that allow appropriate levels of RGS4 synthesis. Under activated conditions (“+Bic,” right), STAU2 condensates disperse into smaller complexes, relieving repression and promoting robust production of nascent RGS4 peptides in dendritic spines.

To test whether STAU2‐mediated translational repression is regulated by synaptic activity, we first asked how synaptic activity influences the distribution of endogenous STAU2 granules in dendrites. Synaptic activity was bidirectionally manipulated in cultured rat hippocampal neurons using pharmacological cocktails that produced either elevated (“+Bic”) or silenced (“+TTX”) synaptic conditions [58, 59, 60, 61] (see Methods). Under resting conditions, only a small fraction of endogenous STAU2 puncta colocalized with the postsynaptic scaffold protein Homer1c, which marks excitatory postsynaptic sites (Figure 9A, boxed regions; Figure 9B). Elevating synaptic activity (+Bic) significantly increased the proportion of STAU2 puncta at Homer1c‐positive postsynaptic regions (Figure 9A,B), while reducing STAU2 puncta number per unit length of dendrite (Figure S4B,C). In contrast, synaptic silencing (+TTX) did not significantly alter STAU2 puncta number, but significantly increased puncta size along dendrites (Figure S4B,C), while decreasing the proportion of STAU2 puncta localized at postsynaptic sites (Figure 9A,B). Thus, elevated synaptic activity disperses dendritic STAU2‐RNPs and promotes their recruitment to synapses, whereas synaptic silencing promotes the enlargement of dendritic STAU2 condensates and reduces their synaptic delivery.

We next assessed whether these synaptic activity‐dependent bidirectional changes in the assembly state of STAU2 granules affect the synaptic translation of its cargo mRNA. We addressed this by using a SunTag translational reporter system for *RGS4* mRNA, in which local translation is reported by GFP fluorescence bound to tandem SunTag epitopes in the nascent peptide (Figure 9C). No specific SunTag puncta were detected above background in the absence of doxycycline (‐Dox), whereas SunTag‐GFP puncta were robustly induced upon doxycycline addition (+Dox), confirming the specificity of the inducible reporter system (Figure S4D). Acute treatment with the translation inhibitor puromycin (+Puro) for 10 min markedly reduced the SunTag‐GFP puncta signal, demonstrating that these puncta depend on ongoing translation (Figure S4E). In control neurons, elevated synaptic activity robustly increased SunTag fluorescence in dendritic spines (Figure 9D, “+Bic”, arrowheads; Figure 9E). Conversely, silencing synaptic activity greatly reduced translation efficiency (Figure 9D, “+TTX”; Figure 9E), indicating that bidirectional manipulation of synaptic activity produces reciprocal changes in *RGS4* translation levels. We next assessed whether this activity‐dependent translational control depends on STAU2. In neurons overexpressing STAU2 (Stau2 OE), the basal SunTag signal under resting conditions was significantly reduced compared with control neurons, and the amplitudes of activity‐induced changes evoked by both synaptic activation and inhibition were blunted (Figure 9E,F, “Stau2 OE”). Similarly, in neurons with endogenous STAU2 knocked down (siStau2), the bidirectional modulation of *RGS4* translation was strongly attenuated (Figure 9E,F, “siStau2”). These results indicate that both excessive and deficient STAU2 levels impair activity‐dependent translational control, demonstrating that an appropriate level of STAU2 is required to mediate fine‐tuned regulation of its target mRNA at synapses.

To assess whether STAU2 gates synaptic plasticity, we quantified spine remodeling following bidirectional manipulation of neuronal activity. Elevated network activity (+Bic) and synaptic silencing (+TTX) oppositely regulated spine morphology, increasing and decreasing the spine width‐to‐length ratio, respectively. Both STAU2 overexpression and knockdown attenuated this response, mirroring its effect on local *RGS4* translation (Figure S4F–H). Thus, proper STAU2 expression levels are required to maintain proper activity‐dependent synaptic structural remodeling.

Collectively, synaptic activity bidirectionally remodels STAU2‐RNP assembly at synapses: increased activity is associated with smaller STAU2 assemblies and enhanced local *RGS4* translation, whereas synaptic silencing favors larger STAU2 condensates and reduced *RGS4* translation.

### 2.11 Neuronal STAU2 Condensates are Upregulated in AD Models

**FIGURE 10:**
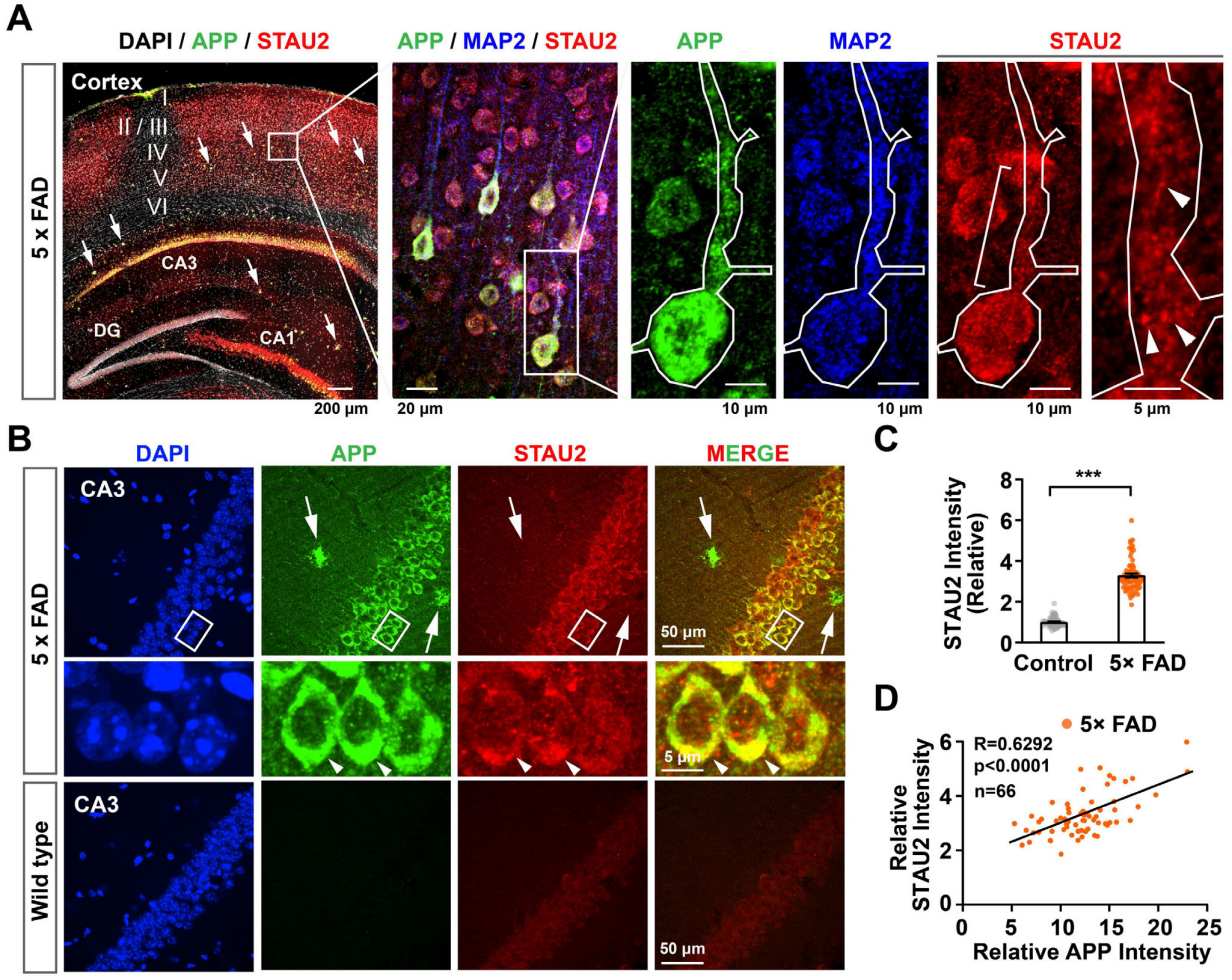
Neuronal STAU2 condensates are upregulated in AD neurons. (A) Representative IF images of brain sections from 4‐month‐old 5×FAD mice showing Aβ plaques (green), STAU2 (red), the dendritic marker MAP2 (blue), and nuclei (DAPI, gray). Aβ plaques are indicated by arrows. Higher‐magnification views of layer 3/4 cortical neurons are shown in the middle panels, with dendritic STAU2 “aggregates” indicated by arrowheads in the right panels. Scale bars: 200 µm (left), 20 µm (middle), 10 µm (right). (B) Representative images of brain sections from 5×FAD mice and age‐matched controls showing STAU2 (red) and Aβ plaques (green, arrows). STAU2 forms intracellular aggregates that colocalize with APP (arrowheads). Scale bars: 50 µm. (C) Quantification of endogenous STAU2 intensity in hippocampal CA3 neurons from 5×FAD mice and age‐matched wild‐type controls. Data are mean ± SEM (*n* = 70 [Control] and 66 [5×FAD] neurons; 3 mice per group), ^***^ *p* < 0.001, Student's *t*‐test. (D) Scatter plot of endogenous STAU2 intensity vs. APP intensity in hippocampal CA3 neurons of 5×FAD mice. Linear regression (black line) shows a strong positive correlation (Pearson's *r* = 0.6292, *p* < 0.0001; *n* = 66 neurons from 3 mice).

Accumulating evidence underscores that the pathological transition of RNA condensates into solid‐like aggregates is a key contributor to neurodegeneration [62]. To determine whether aberrant STAU2 condensates also form in diseased neurons, we examined endogenous STAU2 expression in cortical and hippocampal neurons from 4‐month‐old 5×FAD mice, an Alzheimer's disease model, at an age at which Aβ‐plaque pathology is prominent in both regions (Figure 10A, arrows). Co‐staining STAU2 with the dendritic marker MAP2 revealed STAU2 aggregates in both the soma and apical dendrites of layer‐3/4 cortical neurons (Figure 10A, arrowheads). Notably, STAU2 was not detectable within Aβ plaques themselves (Figure 10B, arrow), but instead colocalized with intracellular APP in morphologically intact neurons (Figure 10B, arrowheads). Quantification of hippocampal CA3 neurons showed that overall STAU2 levels were significantly increased in 5×FAD mice compared with age‐matched wild‐type controls (Figure 10B,C). Moreover, in intact CA3 neurons of WT mice, both STAU2 and APP intensities were low, but STAU2 intensity was positively correlated with APP intensity (Figure S5; Pearson's *r* = 0.37, *p* < 0.01), whereas in 5×FAD CA3 neurons, in which both expression levels were significantly increased, this correlation was markedly strengthened (Figure 10D; Pearson's *r* = 0.63, *p* < 0.0001). These findings indicate that STAU2 forms dendritic aggregates in diseased cortical and hippocampal neurons in an AD model and that STAU2 levels are tightly associated with APP accumulation in both healthy and diseased neurons.

## 3 Discussion

**FIGURE 11:**
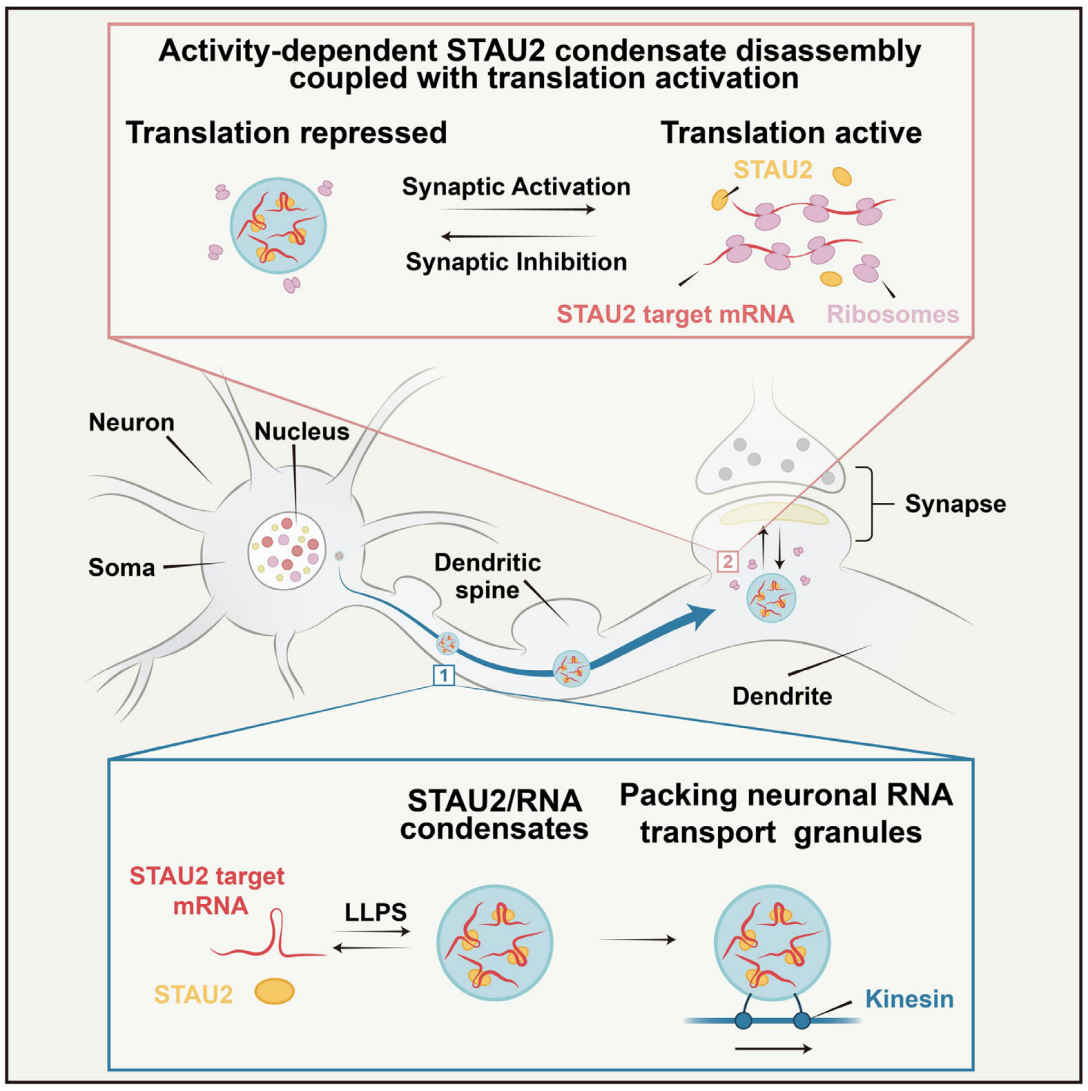
Model diagram illustrating how STAU2 modulates dendritic development in neurons by regulating mRNA transport and translation through liquid–liquid phase separation.

Emerging evidence indicates that numerous RNA‐containing cellular granules are membraneless condensates assembled via phase separation of RNA‐binding proteins [1, 5, 63, 64, 65, 66, 67]. As a key regulator of cortical development, the dsRNA‐binding protein STAU2 controls the metabolism of thousands of RNAs, especially their transport and local translation in neuronal dendrites. However, the mechanism by which STAU2 assembles various RNAs into RNPs remains elusive. In this study, we demonstrate that STAU2 undergoes concentration‐dependent phase separation to form a dynamic, liquid‐like condensate in developing dendrites. STAU2 condensate recruits and enriches target mRNAs (e.g., *RGS4*, *CaMKIIα*), assembling them into transport granules, thus enabling efficient RNA delivery to distal dendrites via the kinesin motor (Figure 11). The RNA‐embedded STAU2 condensates transition from a liquid to a gel state, stabilizing the internal RNAs while repressing their translation during dendritic transport [26, 27]. Our data further demonstrate that synaptic activity bidirectionally remodels STAU2 condensates in parallel with changes in local translation of STAU2‐associated mRNAs, supporting a model in which the condensation state of STAU2 tunes mRNA availability and translation in response to neuronal activity [68]. Consistent with recent models of granule reorganization, such remodeling may involve coordinated interactions between STAU2‐RNPs and other synaptic RNA‐binding proteins, including FMRP, PUM2, and DDX6 [29, 61, 66], fine‐tuning mRNA availability and translation in response to neuronal activity.

Previous work proposed a “patrolling/sushi‐belt” model in which STAU2 and the *RGS4* 3**'**UTR mediate dendritic transport and activity‐dependent synaptic recruitment of *RGS4* mRNA [21]. Our findings extend this model by identifying a downstream step after recruitment: activity‐dependent remodeling of STAU2 granules promotes *RGS4* mRNA release and local translation in dendritic/synaptic compartments. These results support a multistep framework linking STAU2‐dependent mRNA delivery to activity‐coupled translational activation. Such spatially restricted translation may confer synapse specificity and temporal precision to plasticity‐related protein synthesis. **S**ynaptic activity may indirectly remodel STAU2 condensates via activity‐dependent signaling pathways rather than through direct Ca^2^^+^ sensing by STAU2, which lacks a canonical Ca^2^^+^‐binding domain. Consistent with this possibility, Ca^2^^+^/CaMKII signaling has been shown to regulate condensate assembly at synapses [69, 70]; thus, activity‐regulated kinases such as CaMKII may modulate STAU2‐RNP dynamics by phosphorylating STAU2 or its associated components, a mechanism that warrants future investigation.

Interestingly, the LLPS‐deficient STAU2 mutants impaired distal delivery of *RGS4* mRNA more severely than that of *CaMKIIα* mRNA, suggesting transcript‐specific reliance on trafficking via STAU2 RNPs. Given that the STAU2 ΔRBD2 and 4YE mutants exhibit stronger binding to *RGS4* mRNA than to *CaMKIIα* mRNA (Figure 4F,G), it is unlikely that their residual RNA‐binding capacity remains sufficient for *CaMKIIα* transport but not for *RGS4*. One possibility is that *CaMKIIα* mRNA retains access to other trafficking carriers when STAU2 condensation is impaired. FMRP and CPEB are two key regulators that bind to the 3’UTR of *CaMKIIα* mRNA to facilitate its transport and local translation in dendrites [71, 72]. Whereas STAU2 preferentially controls the synaptic‐activity‐dependent localization of an intron i16‐containing *CaMKIIα* mRNA subpopulation to distal dendrites [23].

As a concentration‐dependent process, phase separation renders protein abundance a critical regulator of its associated cellular functions [42, 73]. Our study demonstrates that STAU2 maintains a homeostatic expression level to safeguard neurogenesis and corticogenesis by spatiotemporally assembling thousands of RNAs into functionally distinct granules [11, 14, 18, 19, 21, 26]. These STAU2‐RNPs represent a balanced outcome of STAU2‐RNA interaction, with protein and RNA levels maintained within appropriate concentrations to ensure the essential dynamics required for efficient motor‐driven transport in the developing dendrites. Conversely, ectopic overexpression of STAU2 leads to the formation of oversized, low‐mobility RNPs that fail to be efficiently transported distally. This ultimately results in impaired dendritic elongation and increased local translation of untransported RNAs, thereby promoting excessive branching. Therefore, our study reveals that maintaining a balanced level of STAU2 phase separation is crucial for preserving proper RNP dynamics and ensuring a coordinated supply of mRNA to the growing tips of neuronal dendrites.

Our findings suggest that dendritic STAU2 granules undergo activity‐dependent bidirectional remodeling, which is coupled to dynamic regulation of local translation of synaptic plasticity‐related factors such as RGS4. This structural remodeling stems from a polymer‐level reorganization, from homopolymeric STAU2 assemblies into a heteropolymeric STAU2‐RNA network, though how this architectural shift senses and responds to fluctuation in synaptic activity remains elusive. These results further support the emerging paradigm that the condensation properties of neuronal RNA granules are under precise activity‐dependent control [61, 68], enabling fine‐tuned local protein synthesis that underpins synaptic plasticity and homeostasis. Beyond physiology, we identify a potential mechanistic convergence between dysregulated STAU2 condensation and neurodegenerative pathology. In AD (5×FAD) models, STAU2 forms dendritic aggregates in cortical and hippocampal neurons. The accumulation of these assemblies is associated with the pathogenesis of these age‐related diseases [62], suggesting that a loss of STAU2 condensate dynamics may be a pathologically relevant event. This phenomenon aligns with a broader theme in neurological disorders, where RBPs undergo a pathogenic liquid‐to‐solid transition. For instance, mis‐splicing induces irreversible aggregation of CPEB4, disrupting synaptic translation and plasticity in autism spectrum disorders [74]; Oxidized DJ‐1 co‐aggregates with α‐synuclein in Parkinson's disease [75]; and aberrant aggregation of FMRP, TDP‐43, or FUS leads to neurodevelopmental defects associated with fragile X syndrome, ALS, and FTD, respectively [76, 77, 78, 79]. Disease‐associated STAU2 aggregates may represent a pathological extension of the activity‐ and RNA‐regulated STAU2 condensation mechanism described here. However, our data are correlative and do not establish whether these assemblies act as drivers of pathology, arise as a consequence of broader proteostatic stress, or reflect a protective response; determining whether manipulation of STAU2 condensation state can ameliorate APP‐associated phenotypes will be an important direction for future studies. A critical unanswered question is how canonical pathological aggregates, such as Aβ, mechanistically engage and remodel STAU2 condensates. Elucidating this relationship will be essential for understanding proteopathic stress converges with RNP dysfunction in age‐related cognitive decline.

Though both STAU1 and STAU2 exhibit phase separation properties, they exert markedly divergent effects on mRNA metabolism. Our study reveals that STAU2 condensate selectively recruits *RGS4* and *CaMKIIα* mRNAs via their 3'UTR, leading to condensate gelation and translational suppression. In contrast, prior work has demonstrated that STAU1 condensate recruits *MTOR* mRNA at its 5'UTR to promote translation, leading to mTOR hyperactivation and dysfunction of the autophagy‐lysosome pathway [73]. STAU1 also recognizes 3'UTR of *MyoD* mRNA to inhibit its translation [80], and targets 3'UTR of *PCP2* mRNA to promote its degradation [81]. The precise mechanisms by which STAU1/2 selectively recruit distinct mRNA cohorts and determine their fates remain an open question and warrant further investigation. Notably, the conserved SSM‐mediated homodimerization [82] and heterodimerization of STAU1 and STAU2 are crucial for Staufen‐mediated decay (SMD) [83]. Therefore, a compelling future direction is to explore whether and how STAU1 and STAU2 condensates functionally coordinate, potentially through co‐condensation or cross‐regulation, to orchestrate integrated RNA metabolic processes such as SMD.

## 4 Experimental Procedures

### 4.1 Plasmids

For expression in the *E. coli* system, the coding sequence of various human STAU2 (Uniprot ID: Q9NUL3) fragments were subcloned into a modified version of the pET32a vector as previously described [84], which contains a Trx‐His6 tag followed by a Precision protease cutting site at the N‐terminus. For expression in mammalian cells, GFP‐STAU2 and various mutant plasmids were generated by cloning the coding sequence of STAU2 into the pEGFP‐C3 vector. For the dual‐luciferase reporter assay, the segment from 984 to 1618 nt of rat *RGS4* 3'UTR (NM_017214.1) containing Staufen‐binding sequences was subcloned into a modified version of the pRF vector, which resulted in the plasmid together with an SV40 promoter, luciferase ORF, a stop code, 984–1618 nt of rat *RGS4* 3'UTR, and SV40 poly(A) tail. MS2‐related plasmids (pCR4‐24XMS2SL‐stable Addgene #31865 and pEFS‐MCP‐SNAP‐NLS plasmid) were kindly provided by Prof. Hanhui Ma (ShanghaiTech University). For RNA reporter constructs, the MS2‐*RGS4* 3'UTR construct was generated by cloning the sequence of an array of 24 unique MS2 hairpins and 984–1618 nt of the rat *RGS4* 3'UTR (NM_017214.1) into a modified version of the pcDNA3.1 vector, resulting in a plasmid containing a CMV promoter, together with HA tag as ORF, a stop codon, MS2 array, a stop codon, 984–1618 nt of rat *RGS4* 3'UTR, and poly(A) tail. The MS2‐CaMKIIα intron16 construct was generated by cloning the sequence of the CaMKIIα long isoform ENSMUST00000102888.9 intron16 [23] and an array of 24 unique MS2 hairpins into a modified version of the pcDNA3.1 vector, resulting in the plasmid containing a CMV promoter, together with CaMKIIα isoform intron16, HA tag as ORF, a stop codon, MS2 array, a stop codon, and poly(A) tail. All the mutations used in this study were generated using the standard PCR‐based mutagenesis method and confirmed by DNA sequencing.

### 4.2 Culture and Transfection of Primary Neuron

Primary hippocampal neurons from Sprague Dawley rat embryos at embryonic day 17 were dissociated and cultured following the previously described protocol [85]. Briefly, coverslips or glass‐bottom dishes (#D29‐20‐1.5‐N, Cellvis) were coated overnight with 0.5 mg/mL poly‐L‐lysine in borate buffer (1 M, pH 8.5). Hippocampal neurons were isolated, dissociated using 2.5% trypsin, and then seeded onto the coated dishes at a density of 3 × 10^4^ cells/cm^2^. The cells were cultured in Neurobasal Medium supplemented with 2% B27 and 1% L‐GlutaMax at 37°C with 5% CO_2_. Between 6–9 days in vitro (DIV), neurons were transfected with Lipofectamine 2000 or 3000 according to the manufacturer's instructions. Live‐cell imaging was performed 24–72 h after transfection. For total RNA labeling experiments, 0.2 nmol CY5‐UTP (#B8333, ApexBio) and 1 µg of the indicated plasmid were mixed and co‐transfected into DIV 6–9 neurons. On DIV 7–10, live‐cell imaging was performed using a Nikon TI2‐E inverted microscope with a Yokogawa spinning confocal disk head (CSU‐W1), equipped with a 40 × 1.3 NA objective. For STAU2 knockdown experiments, 40 pmol siRNA was transfected into each well of a 29 mm glass‐bottom dish using Lipofectamine 2000. Live‐cell imaging was performed 48 h after transfection. The following two STAU2 siRNA oligonucleotides were used to target STAU2 mRNA: siSTAU2‐2: 5′‐gatatgaaccaaccttcaa‐3′; siSTAU2‐3: 5′‐gccagggaactcctaatgaat‐3′.

### 4.3 Culture and Transfection of Rat PC12 Cells

Rat PC12 cells were cultured in high glucose DMEM (#SH30243.01, Hyclone) supplemented with 5% fetal bovine serum (#10091148, Gibco), 10% horse serum (#30074.03, Hyclone), and 1 mM Penicillin/Streptomycin (#SV30082.01, Hyclone) at 37°C with 5% CO_2_. Before seeding, culture dishes were coated with 0.5 mg/ml poly‐L‐lysine in borate buffer (1 M, pH 8.5) for 30 min. For STAU2 knockdown experiments, 100 pmol siRNA was transfected into each well of a 6‐well dish using Polyethylenimine Linear 40K (#49553937, Maokangbio). Cells were harvested and lysed with RIPA buffer (50 mM Tris‐HCl, pH 7.4, 150 mM NaCl, 1% NP‐40, and 0.25% sodium deoxycholate), 72 h after transfection. Lysates were centrifuged at 15 000 g, 4°C for 10 min. The supernatant was separated using 10% SDS‐PAGE gels, and the knockdown efficiency of STAU2 expression was detected by Western blot.

### 4.4 Immunofluorescence Staining and Live‐Cell Imaging of Cultured Neurons

For immunofluorescence staining, neurons cultured on 18 mm glass coverslips (#1001/18, Assistent) were fixed with 4% PFA and 4% sucrose in PBS for 30 min at room temperature. Then, the fixed cells were washed 3 times with PBS and permeabilized with the antibody dilution buffer (0.1% Saponin, 1% BSA, and 0.2% gelatin in PBS, pH 7.4). Next, the neurons were incubated with primary antibodies diluted in the antibody dilution buffer overnight at 4°C, followed by incubation using Alexa Fluor‐conjugated fluorescent secondary antibodies diluted with the antibody dilution buffer for 1 h in the dark at room temperature. Coverslips were then mounted on glass slides using Fluoroshield histology mounting medium (#F6182, Sigma). Confocal imaging of fixed cells was performed on a Nikon TI2‐E inverted microscope with a Yokogawa spinning confocal disk head (CSU‐W1) and a 60 × 1.4 numerical aperture (NA) objective lens. For neuronal analyses, dendrites were identified based on positive immunofluorescence staining for the dendritic marker MAP2. For the dendrite length analysis, a Zeiss Axio Observer Z2 microscope and a 40 × 1.3 NA objective lens was used.

Live‐cell imaging was performed in the live‐imaging medium (15 mM HEPES, 145 mM NaCl, 5.6 mM KCl, 2.2 mM CaCl_2_, 0.5 mM MgCl_2_, 5.6 mM D‐glucose, 0.5 mM ascorbic acid, 0.1% BSA, pH 7.4), which was prewarmed and balanced in the CO_2_ incubator before being added to the cultured neurons. To capture the dynamic movements of STAU2 granules, a Nikon TI2‐E inverted microscope with Yokogawa spinning confocal disk head (CSU‐W1) and a 40 × 1.3 NA objective was used to acquire time‐lapse images of the dendritic regions of the live neurons at a time interval of 6–10 s for 15 min. To capture the dynamic movements of STAU2 granules and RNA granules, live‐cell imaging experiments were conducted using a Leica Thunder microscope equipped with 63 × 1.4 NA objective at a time interval of 2 s for 15 min. The acquired time‐lapse images of live neurons were then processed and analyzed using ImageJ2 (v2.3.0/1.53f, NIH). To monitor the movements of individual RNA and STAU2 granules, the Multi‐Kymograph plugin was used to generate kymographs of these granules along the dendrites.

### 4.5 Trafficking Analysis for STAU2 Granules and RNA Granules

The acquired time‐lapse images of granule movement in apical dendrites of live neurons were processed and analyzed using ImageJ2 (v2.3.0/1.53f, NIH). The TrackMate plugin (v7.11) [86] was used to automatically capture the trafficking speed of moving CY5‐labelled RNA granules, following the steps as previously described [87]. Briefly, in STAU2 knockdown neurons, CY5‐labelled RNA granules were tracked using TrackMate, with the estimated object diameter set to 1 µm and a quality threshold of 2. Then, the detected spots were traced by a simple LAP tracker (linking max distance 6 µm; track segment gap closing 5 µm and 2 frames), with the resulting tracks further filtered by duration longer than 2 consecutive frames. All the output data were statistically analyzed and illustrated in the Prism (v8.2.1, GraphPad).

### 4.6 Culture and Transfection of HEK293T and COS7 Cells

Human HEK293T and COS7 cells were cultured in Dulbecco's modified Eagle's medium (DMEM; gibco) containing 10% Fetal Bovine Serum (FBS), penicillin, and streptomycin at 37°C in a 5% CO_2_ atmosphere at constant humidity [42]. For cell transient transfection, when the cell density reaches 60%–70%, the various plasmids were mixed together with polyethylenimine (PEI) transfection reagent at a ratio of 1:3, and the mixtures were incubated for 20 min, and then transfected into the cells.

### 4.7 Cell Imaging and Data Analysis

COS7 cells were cultured in 6‐well cell culture dishes which has been added a glass coverslip to each well beforehand. When the cell density reached 60%–70%, COS7 cells were transfected with a plasmid encoding GFP‐STAU2 or its mutants. The medium was replaced 6–8 h after transfection, and the cells were cultured for an additional 18–36 h. Cells were fixed in 4% PFA, permeabilized with PBS containing 0.2% Triton X‐100, stained with DAPI, and processed for fluorescence microscopy imaging (Leica TCS SP5). For puncta‐counting, images were collected from 4 to 6 independent areas randomly selected under the microscope for the puncta counting. In each group of experiments, at least 600 cells that were able to emit fluorescence normally were counted, and cells with more than two distinct fluorescent puncta were considered to be puncta‐positive cell. Each set of experiments was performed using a single‐blind method, and the images were processed using ImageJ software.

### 4.8 Western Blotting

Human HEK293T cells and rat hippocampal neurons were transiently transfected or co‐transfected with GFP‐STAU2 or its mutant plasmids, dual‐luciferase reporter plasmid, or siRNA with polyethyleneimine transfection reagent (Poly‐sciences). Human HEK293T cells were harvested 36 h post‐transfection and lysed in a buffer containing 50 mM Tris (pH 7.4), 150 mM NaCl, 1% Nonidet P‐40, and protease inhibitor cocktail (Thermo, A32955). Various lysates were added to the SDS‐PAGE loading buffer and mixed thoroughly, samples were boiled at 105°C for 10 min, centrifuged at high speed to remove debris, and then subjected to SDS‐PAGE. Proteins were transferred to a 0.45 µm polyvinylidene difluoride (PVDF) membrane (Millipore) for 2 h in the ice bath. The membrane was blocked with 5% bovine serum albumin in TBST buffer containing 20 mM Tris‐HCl (pH 7.4), 150 mM NaCl, and 0.1% Tween‐20 at room temperature for 1 h and incubated with mouse anti GFP‐Tag mAb (ABclonal, AE012) at a 1/4000 dilution at 4°C overnight. After three 15‐min washes with TBST buffer, the membrane was incubated with horseradish peroxidase‐conjugated goat anti‐mouse antibody (ABclonal, AS003) at a 1/5000 dilution at room temperature for 1 h. Following three additional washes, protein bands were visualized on a LAS4000 Chemiluminescence Imaging System.

### 4.9 Protein Expression and Purification

Recombinant proteins were expressed in *E. coli* BL21 (DE3) host cells in the LB medium at 37°C. Until OD_600_≈0.7–0.8, the bacterial solution was cooled at 4°C for 20–30 min and subsequently added with IPTG to a final concentration of 0.3 mM to induce protein expression at 16°C for 16–18 h. The cells were collected by centrifugation and resuspended in the buffer containing 20 mM Tris‐HCl (pH 8.0), 500 mM NaCl, 5 mM imidazole, and then lysed by sonication, and the cell debris was removed by high‐speed centrifugation at 39 190 × g for 30 min at 4°C. The supernatant was purified using a Ni^2+^‐NTA agarose affinity column followed by SEC (using HiLoad 26/600 superdex 75/200 pg columns on an AKTA FPLC system, GE Healthcare) with buffer containing 50 mM Tris‐HCl (pH 8.0), 500 mM NaCl, 2 mM DTT.

### 4.10 In Vitro Transcription and RNA Labelling

In vitro transcription was performed using the RiboMAX Large Scale RNA Production Systems (Promega) for in vitro transcription of RNA according to the manufacturer's instructions. The DNA template must contain a unique T7 sequence and is generated by PCR amplification using T7‐forward primer and reverse primer, purified by agarose gel electrophoresis. Transcription reaction (50 µL) contains 5 µg DNA template, 15 µL of rNTPs (25 mM ATP, CTP, GTP, UTP), 5 µL Enzyme Mix, and 10 µL T7 Transcription 5 × buffer. RNA for in vitro translation assay, replacement of GTP with 1/10 of Ribo m7G Cap Analog (Promega) in the transcription reaction to mimic the mRNA 5' end‐cap structure to improve the translation efficiency of mRNA. The transcription reaction was carried out at 37°C for 4 h and followed by the addition of RQ1 RNase‐Free DNaseI (Promega) to digest the excess DNA template at 37°C for 30 min, added 2 × RNA loading buffer (denaturing) and boiling at 95°C for 10 min, followed by placing on ice for 5 min, further purified by urea‐denaturing PAGE. RNA was finally precipitated with 2.5 times the volume of anhydrous ethanol and 1/10 of a volume 3 M NaAc (pH 5.3), dissolved and stored in RNase‐Free water or the indicated buffer.

For RNA fluorescent labelling, the transcription reaction was added to 5‐amino‐allyl UTP (Biotium) at 1:4 ratio (amino allylUTP: UTP), and the subsequent procedure was referred to as the in vitro transcription. The fluorophore Cy3 NHS ester (AAT Bioquest) was mixed well in a buffer containing 100 mM NaHCO_3_ (pH 8.3) at a molar ratio of 1:1 with the RNA and incubated for 1 h at room temperature, protected from light. The Cy3‐labelled RNA was purified as previously described.

### 4.11 Electrophoretic Mobility Shift Assay (EMSA)

EMSA was carried out as previously described [88]. Preparation of 1% agarose gel with 0.5 × TBE containing 1/10 000 SYBR Gold (Thermo Fisher Scientific). The 20 ng/µL of *RGS4* 3'UTR mRNA or *CaMKIIα* intron16 mRNA was incubated with different concentrations of indicated proteins for 10 min on ice. The reactions were added an equal volume of 2 × RNA loading buffer (non‐denaturing) and subjected to a 1% agarose gel in 0.5 × TBE run at 100V constant current for 50 min at 4°C. The agarose gel was detected using the SYBR Gold option in ChemiDocTM MP Imaging System (Bio‐Rad).

### 4.12 In Vitro Liquid–Liquid Phase Separation (LLPS) Assay

For protein fluorescent labelling, the freshly purified STAU2 was incubated at a molar ratio of the fluorophores iFluor 488 NHS ester (AAT Bioquest) to protein at 1:1 at room temperature and protected from light. The reaction was quenched by adding pre‐prepared 2 M Tris‐HCl (pH 8.3) to a final concentration of 200 mM, then purified by Hitrap desalting column (HiPrep 26/10 Desalting on an AKTA FPLC system, GE Healthcare) with buffer containing 50 mM Tris‐HCl (pH 8.0), 500 mM NaCl, and 2 mM DTT.

Before the assays, the purified proteins were centrifuged at 15 871 × g for 10 min at 4°C to remove protein aggregates and other debris. All in vitro phase separation experiments were performed in a buffer A containing 50 mM Tris‐HCl (pH 8.0), 100 mM NaCl, 2 mM DTT, excluding the crowding agent (polyethylene glycol, PEG) unless mentioned in the figure description, and the resulting liquid–liquid phase separation phenomenon was characterized by sedimentation‐based methods or microscopic imaging.

For sedimentation‐based analysis, the proteins were mixed with the buffer at the indicated protein concentration and salt concentration in the tubes. The reaction was carried out at room temperature for 10 min, followed by centrifugation at 25°C for 10 min at 18 407 × g. The supernatant was aspirated from the centrifuged sample and transferred to a new tube (Supernatant is marked as “S”). The precipitate was washed three times and mixed with an equal volume of buffer A (Pellet is denoted as “P”). The samples were added an equal volume of 2 × SDS‐PAGE loading buffer and mixed thoroughly, boiled at 105°C for 10 min, and subsequently identified by SDS‐PAGE. The intensities of protein bands stained with Coomassie blue staining were quantified by Adobe Photoshop. Pellet percentage (%) = Pellet band intensity / (Pellet band intensity + Supernatant band intensity) ×100.

For microscopic imaging analysis, the protein with or without RNA (fluorophore‐labeled or unlabeled) mixtures were first reacted in the tube, then injected into a home‐made glass flow chamber. After a period of time, the droplets formed by the phase separation were observed using a differential interference contrast (DIC) microscope (Olympus IX73) or a confocal microscope (Leica TCS SP8). The size of the droplets was quantified using the ImageJ software.

### 4.13 FRAP Assay and Data Analysis

For living cell FRAP assays, cells were cultured in glass‐bottom dishes and transfected as described above. Living cells with two or more puncta and a puncta diameter of approximately 1–2 µm were selected for the FRAP experiments, and living cells FRAP were performed on a confocal microscope (Leica TCS SP8), and the GFP signal was photobleached using a 488 nm laser beam. The value of the fluorescence intensity difference between prebleach and postbleach was defined as 100%, and the fluorescence intensity in the photobleaching region was recorded by the instrument software and normalized to the initial intensity before bleaching.

For the in vitro FRAP analysis, the iFluor 488 signal of STAU2 droplets was bleached using a 488 nm laser beam with a Leica TCS SP8 confocal microscope at room temperature. The fluorescence intensity difference between prebleaching and at time 0 (the time point right after the photobleaching pulse) was normalized to 100%. The experimental control is to quantify fluorescence intensities of similar droplet regions without photobleaching. All image intensity was measured by Mean ROI and further analyzed by GraphPad Prism 8.

The FRAP assay was performed using DIV10 rat hippocampal neurons transfected with EGFP‐STAU2 plasmids 24 h prior. Briefly, the culture medium was replaced with prewarmed imaging buffer, and the glass‐bottom dish containing neurons was placed on a Nikon TI2‐E inverted microscope equipped with a Yokogawa CSU‐W1 spinning disk confocal head. Imaging was conducted using a 60 × 1.4 NA oil objective. Time‐lapse images of EGFP‐STAU2‐expressing neurons were acquired at 1‐s intervals. After capturing six baseline images, the GFP signal was photobleached using a 488 nm laser at 90% maximal intensity for 200 ms. Following photobleaching, fluorescence recovery was monitored for 5 min, with images acquired every second. Time‐lapse images were processed and analyzed using Nikon Elements AR software. FRAP efficiency was then calculated as:

$$E=\frac{\left(Ft-F0\right)}{\left(Fc-F0\right)\;}\times 100\%$$

Ft: Fluorescence intensity at time *t*; F0: Fluorescence intensity immediately after photobleaching; Fc: Corrected fluorescence intensity before photobleaching (adjusted using an exponential one‐phase decay fit in GraphPad to account for system bleaching); All output data were statistically analyzed and visualized using Prism (v8.2.1, GraphPad).

### 4.14 Dual‐Luciferase Reporter Assay

HEK293T cells were cultured in 6‐well cell culture dishes and when the cell density reached 60%–70%, the Firefly luciferase reporter plasmid (control luciferase or luciferase‐*RGS4* 3'UTR plasmid, 1100 ng) and Renilla luciferase internal plasmid (100 ng), together with the GFP‐STAU2 and its deletion or mutant plasmid (800 ng), were co‐transfected with HEK293T cells using polyethyleneimine (PEI) transfection reagent (Poly‐sciences) according to the manufacturer's protocol with the slight modifications. After 36 h, Firefly expression and Renilla luciferase expression were measured using the Dual Luciferase Reporter Assay System (Promega) according to the manufacturer's instructions, and GFP‐STAU2 protein expression was detected by western blotting as previously described.

### 4.15 In Vitro RNA Stability Assay

Recombinant STAU2, STAU2 ∆RBD2, and STAU2 4YE proteins (10 µM) were incubated with *RGS4* mRNA (20 ng/µL) for 10 min at room temperature. The mRNA‐protein complexes were then treated with RNase A for 10 min at room temperature. After digestion, *RGS4* mRNA was extracted using phenol:chloroform:isoamyl alcohol (25:24:1) and analyzed by nondenaturing agarose gel electrophoresis.

### 4.16 In Vitro Translation and Luciferase Detection Assay

In vitro translation of luciferase mRNA was performed using the Flexi Rabbit Reticulocyte Lysate System (Promega). *Luciferase* mRNA was denatured at 65°C for 3 min and immediately cooled on ice. Following the manufacturer's protocol with slight modifications, each component of the in vitro translation reaction buffer (50 µL) was added into the RNase‐Free tube in the following order: 33 µL of Flexi Rabbit Reticulocyte Lysate, 0.5 µL of amino acid mixture minus leucine (1 mM), 0.5 µL of amino acid mixture minus methionine (1 mM), 1 µL of RNasin Ribonuclease Inhibitor (40 U/µL, Thermo Fisher Scientific), 7 µL of Nuclease‐Free water, 7 µL of different concentrations of protein in the buffer containing 50 mM Tris‐HCl (pH 8.0), 500 mM KCl, 2 mM DTT, and 1 µL of luciferase mRNA (1 mg/mL). All in vitro translation reactions are mixed thoroughly, centrifuged briefly and then incubated for 90 min at 25°C in a constant metal bath.

The translation of luciferase was monitored with a standard luciferase assay system (Promega). Briefly, 50 µL of luciferase substrate was added to each well of an opaque 96‐well plate, followed by aspiration of 2 µL of the reacted  in vitro translation reaction, mixing well immediately, and measuring the end‐point luminescence at 25°C using a Synergy H4 Hybrid Multi‐Mode Reader (Agilent BioTek). Luminescence measurements represent mean values obtained from three independent in vitro translation reactions, each of them measured three times. The curve was fitted using the *[Inhibitor] vs response‐Variable slope* formula via GraphPad Prism 8.0.

### 4.17 Brain‐Region and Cell‐Type Analysis of STAU2 mRNA Expression

Publicly available transcriptomic datasets from the Allen Brain Atlas were used to examine *STAU2* expression at both regional and single‐cell resolution. For single‐cell resolution analysis, mouse cortical and hippocampal 10x RNA‐seq data were downloaded from the Allen Cell Types database (10x sequencing dataset: https://celltypes.brain‐map.org/rnaseq/mouse_ctx‐hpf_10x). The dataset was filtered according to annotated neuronal populations, and *STAU2* expression was visualized across selected neuronal subtypes to assess its cell‐type‐specific distribution. Regional expression of *STAU2* mRNA across the mouse sagittal brain was examined using the Allen Mouse Brain Atlas in situ hybridization dataset, reference number 248566 (https://mouse.brain‐map.org/experiment/show?id = 248566). The oligonucleotide probe sequence used for in situ hybridization was 5′‐TCCAAAGCCATTCCCAAA‐3′, which detects *STAU2* transcripts.

### 4.18 SunTag Translation Reporter System

SunTag‐related plasmids (pcDNA4TO‐24×GCN4‐v4‐24×PP7 and pHR‐scFv‐GCN4‐sfGFP‐GB1) were kindly provided by Prof. Weirui Ma (Zhejiang University) [89]. To generate a translation reporter for *RGS4* mRNA, nucleotides 984–1618 of the rat *RGS4* 3′UTR were cloned into the pcDNA4TO‐24×GCN4‐v4‐24×PP7 backbone to generate pcDNA4TO‐24×GCN4‐RGS4_3UTR‐24×PP7. This Tet‐On SunTag reporter system enables doxycycline‐inducible expression of a reporter mRNA carrying tandem GCN4 epitopes and the *RGS4* 3′UTR, allowing nascent translation events to be detected by co‐expressed scFv‐GCN4‐sfGFP. On DIV13, rat hippocampal neurons were co‐transfected with pcDNA4TO‐24×GCN4‐RGS4_3UTR‐24×PP7, pHR‐scFv‐GCN4‐sfGFP‐GB1, and either mCherry‐STAU2 or STAU2 siRNAs. On DIV14, reporter expression was induced with 1 µg/mL doxycycline for 4 h. Only neurons expressing the required SunTag reporter components were selected for analysis. Discrete GFP‐positive puncta above the local dendritic background were scored as SunTag translation puncta. To examine whether these GFP puncta represented active translation events, 100 µg/mL puromycin was added to the live‐imaging medium during continuous live‐cell imaging to inhibit nascent peptide synthesis. Synaptic activity was bidirectionally manipulated using pharmacological treatments. For excitatory conditions (“+Bic”), neurons were treated for 1 h with 50 ng/ml BDNF (PeproTech, 450‐02), 30 µM bicuculline (Abcam, ab120110), and 50 µM 4‐AP (MCE, HY‐B0604) [58, 59, 60]. For silencing conditions (“+TTX”), neurons were treated for 4 h with 1 µM TTX (Shanghai Charm‐Analysis, 20–309500), 50 µM D‐AP5 (MCE, HY‐100714A), and 100 µM DNQX (Abcam, ab120018) [61]. Neurons were then fixed and immunostained with anti‐GFP antibody as previously described [90]. Confocal imaging was performed on a Nikon Ti2‐E inverted microscope equipped with a Yokogawa CSU‐W1 spinning‐disk head and a 60×/1.4 NA oil‐immersion objective.

### 4.19 Neurodegenerative Disease Animal Model

All animal experiments were conducted in accordance with institutional and regulatory guidelines and were approved by the Animal Ethics Committee of ShanghaiTech University (approval number: 20250401001). Four‐month‐old 5×FAD transgenic mice were kindly provided by Prof. Zhenge Luo (ShanghaiTech University) and maintained on a C57BL/6J genetic background. Age‐matched wild‐type C57BL/6J mice were used as controls.

For brain slice staining, brain tissue processing was performed as previously described [90]. Briefly, mouse brains were coronally sectioned into 40‐µm floating sections using a vibratome. Floating sections were incubated with anti‐STAU2 (Proteintech, 15998‐1‐AP), anti‐MAP2 (Aves Labs, MAP), and anti‐APP (BioLegend, 803014) antibodies (1:500) in TBS containing 1% BSA overnight at 4°C, followed by secondary antibodies (1:2500) for 2 h at room temperature. Confocal imaging was performed on a Nikon Ti2‐E inverted microscope equipped with a Yokogawa CSU‐W1 spinning‐disk head and a 20×/0.8 NA oil‐immersion objective.

For biochemical analysis, mouse cortical tissues were homogenized in ice‐cold lysis buffer (50 mM Tris‐HCl, 150 mM NaCl, 1% NP‐40, 0.25% sodium deoxycholate, pH 7.4) supplemented with protease inhibitor cocktail, centrifuged at 15 000 g for 60 min at 4°C. The supernatant was collected as the soluble fraction, and all steps were performed on ice. Aliquots were snap‐frozen in liquid nitrogen and stored until use. For SDS–PAGE and immunoblotting, samples were mixed with 5× SDS sample buffer and boiled at 95°C for 5 min before electrophoresis and Western blot analysis.

### 4.20 Quantification and Statistical Analysis

For all quantifications described above, statistical analyses were performed, and data were plotted using GraphPad Prism8 (GraphPad Software Inc., CA). Data are shown as the mean ± SEM or mean ± SD of the mean from at least three independent experiments. *p*‐values were calculated using an unpaired two‐tailed *t*‐test or one‐way analysis of variance (ANOVA) with Tukey's multiple comparison test as indicated. In the figures, ^∗^ *p* < 0.05, ^∗∗^ *p* < 0.01, ^∗∗∗^ *p* < 0.001, ^∗∗∗∗^ *p* < 0.0001.

## Author Contributions

S.H., Y.C., and R.Z. designed and performed the experiments with support from S.L., Y.F., H.Z., and Y.X. S.H., Y.C., T.W., and W.W. analyzed the data and cowrote the manuscript. W.W. conceived the idea and coordinated the research.

## Conflicts of Interest

The authors declare no conflicts of interest.

## Supporting information




**Supporting File 1**: advs75852‐sup‐0001‐SuppMat.pdf.

**Supporting File 2**: advs75852‐sup‐0002‐movieS1.avi.

**Supporting File 3**: advs75852‐sup‐0003‐movieS2.avi.

**Supporting File 4**: advs75852‐sup‐0004‐movieS3.avi.

## Data Availability

The data that support the findings of this study are available from the corresponding author upon reasonable request.
