# Supplementary material for: Fine‐Tuned Regulation of mRNA Translation and Transport by STAU2 Condensate Facilitates Neuronal Development and Plasticity
Source: Adv Sci (Weinh). 2026 May 29;13(47):e00044. doi: 10.1002/advs.202600044 (PMC13335923; doi:10.1002/advs.202600044)
Supplement: Supplementary file 1 [file ADVS-13-e00044-s004.pdf]

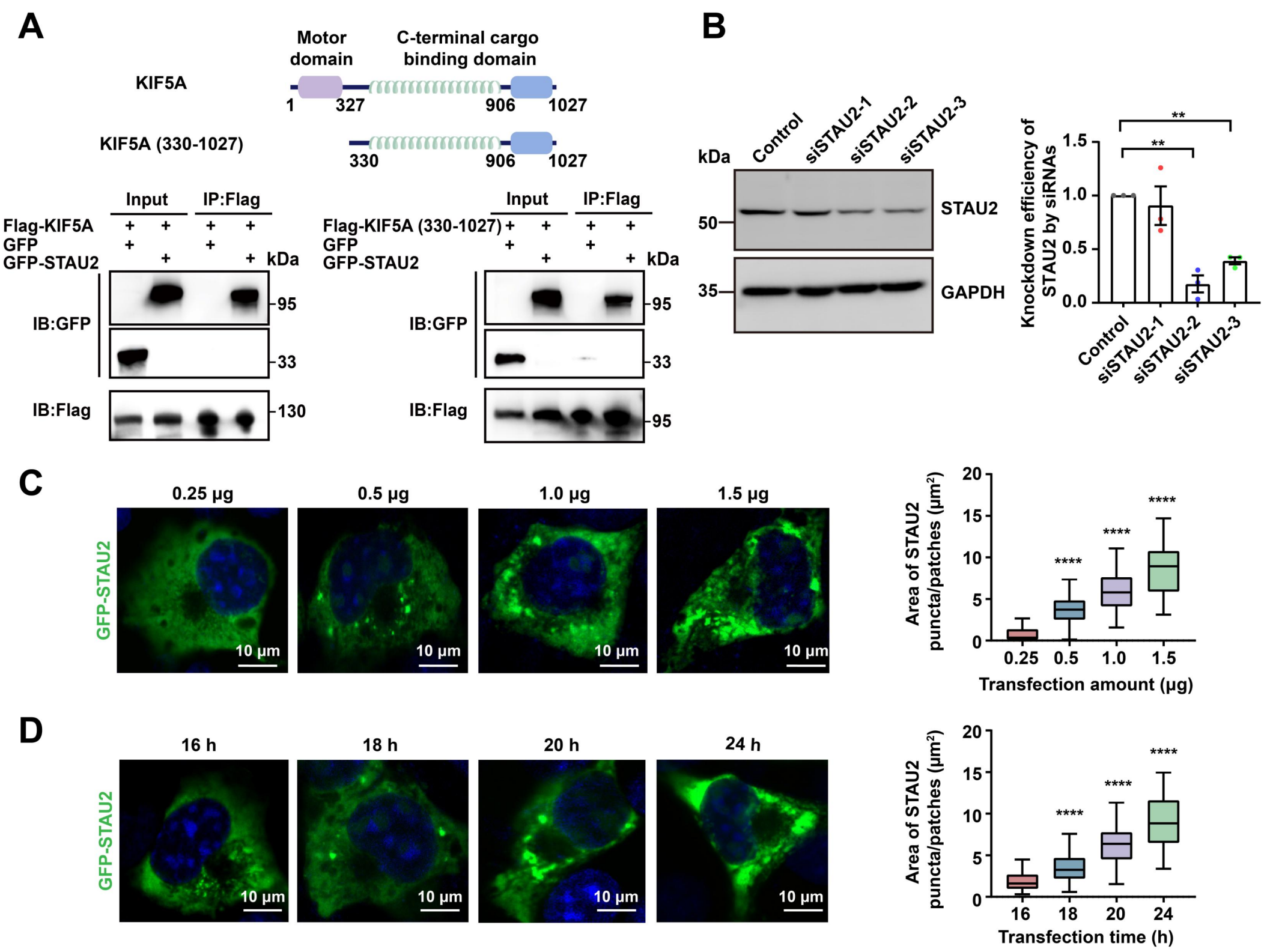

Figure S1. STAU2 forms RNP granules and facilitates RNA delivery in dendrites of primary neurons.

(A) Co-immunoprecipitation (Co-IP) analysis of the lysates from HEK293 cells co-expressing GFP vector or GFP-STAU2 with Flag-KIF5A or Flag-KIF5A (aa 330-1027).

(B) Knockdown efficiency of STAU2 by siRNAs in PC12 cells analyzed by Western blot (left). Quantification of knockdown efficiency of siSTAU2. (right). Data are derived from three independent experiments and are expressed as mean ± SEM, **p <0.01, Student's *t*-test.

(C) Representative fluorescence images of COS7 cells expressing GFP-STAU2 at different levels (left). Quantification of the area of puncta/patches under different amount of STAU2 transfections in COS7 cells (right; 200 puncta-positive cells were counted for each different transfection amount). Scale bars: 10 μm. ****p < 0.0001, Student's *t*-test.

(D) Representative fluorescence images of COS7 cells expressing GFP-STAU2 at different time points (left). Quantification of the area of puncta/patches under different transfections times of STAU2 in COS7 cells (right; 200 puncta-positive cells were counted for each different transfection time). Scale bars: 10 μm. ****p < 0.0001, Student's *t*-test.

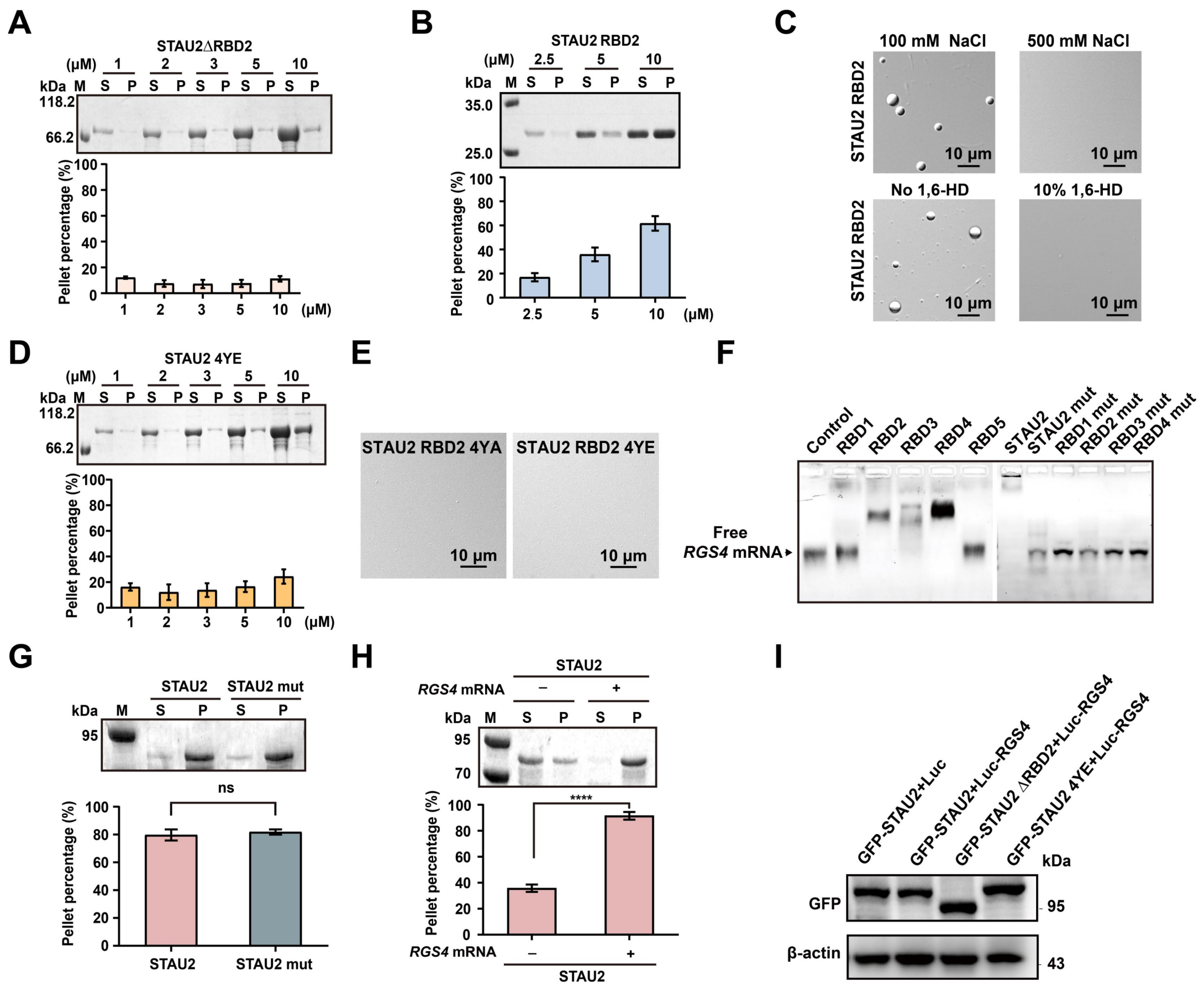

Figure S2. RBD2 is essential for STAU2 phase separation.

(A&B) Sedimentation assay analysis of STAU2 ΔRBD2 **(A)** or STAU2 RBD2 **(B)**. Data are from three independent experiments and are expressed as mean ± SD.

(C) Representative DIC images of STAU2 RBD2 (10 μM) treated with NaCl (upper panel) or 1,6-hexanediol (1,6-HD, lower panel). Scale bars: 10 μm.

(D) Sedimentation assay analysis of STAU2 4YE. Data are from three independent experiments and are expressed as mean ± SD.

(E) Representative DIC images of STAU2 RBD2 4YA and STAU2 RBD2 4YE at 10 μM. Scale bars: 10 μm.

(F) EMSA experiments of *RGS4* 3'UTR mRNA (20 ng/μL) with or without various STAU2 fragments or their mutants (8 μM) in non-denaturing agarose gel.

(G) Sedimentation assay analysis of STAU2 and STAU2 mut (10 μM). Data are from three independent experiments and are expressed as mean ± SD, ns, not significant ($P > 0.05$), Student's *t*-test.

(H) Sedimentation assay analysis of STAU2 (3 μM) in the presence or absence of *RGS4* mRNA (20 ng/μL). Data are from three independent experiments and are expressed as mean ± SD, ****$p < 0.0001$, Student's *t*-test.

(I) Western-blotting analysis showing protein expression of GFP-STAU2 and its mutants in the dual-luciferase reporter assay corresponding to Fig. **5F**.

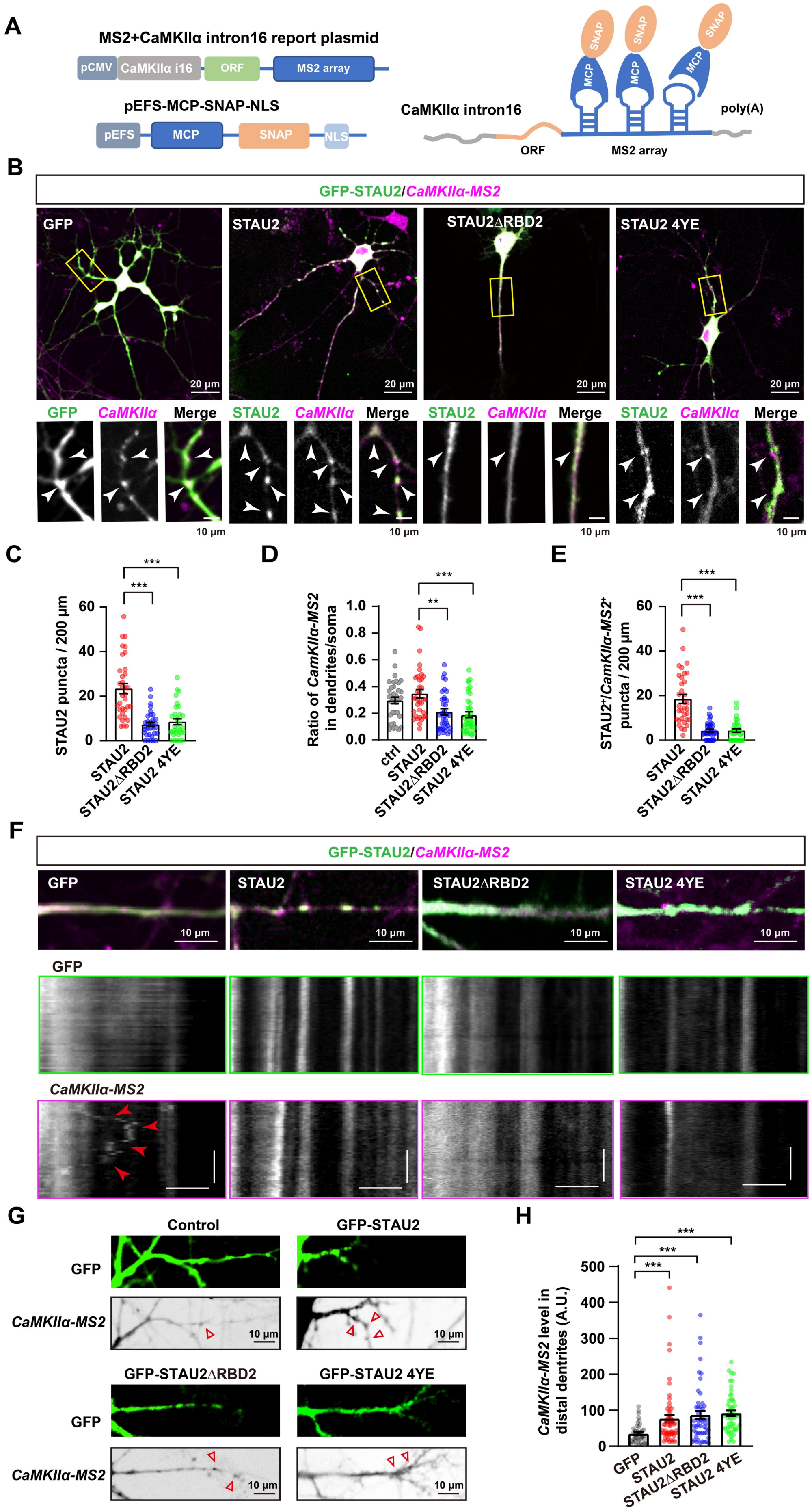
A
MS2+CaMKIIα intron16 report plasmid
pCMV
CaMKIIα i16
ORF
MS2 array
pEFS-MCP-SNAP-NLS
pEFS
MCP
SNAP
NLS
SNAP
MCP
CaMKIIα intron16
poly(A)
ORF
MS2 array
B
GFP-STAU2/CaMKIIα-MS2
GFP
STAU2
STAU2ΔRBD2
STAU2 4YE
20 μm
GFP
CaMKIIα
Merge
STAU2
10 μm
C
STAU2 puncta / 200 μm
D
Ratio of CamKIIα-MS2 in dendrites/soma
ctrl
E
STAU2+/CamKIIα-MS2+ puncta / 200 μm
F
CaMKIIα-MS2
G
Control
GFP-STAU2
GFP-STAU2ΔRBD2
GFP-STAU2 4YE
H
CaMKIIα-MS2 level in distal dendrites (A.U.)

Figure S3. STAU2 granules colocalize with *CaMKIIα mRNA* in dendrites of cultured hippocampal neurons.

(A) Scheme of *CaMKIIα-MS2* and MCP-SNAP expression cassettes (left) and the MS2 mRNA reporter system (right). pEFS (promoter), ORF (open reading frame), NLS (nuclear localization signal), MCP (MS2 coat protein).

(B) *CaMKIIα-MS2* and MCP-SNAP mRNA reporter systems were transfected with GFP-N1, GFP-STAU2, GFP-STAU2 ΔRBD2, or GFP-STAU2 4YE in DIV9 rat hippocampal neurons. Live-cell imaging was performed on DIV10 using spinning disc confocal microscopy. Representative dual-color time-lapse images show the subcellular localization and movement of STAU2 (green) and *CaMKIIα-MS2* (magenta) puncta in dendrites of neurons expressing the indicated constructs. Boxed regions are amplified in the bottom panels, with *CaMKIIα-MS2*-positive puncta (*CaMKIIα* mRNA) indicated by arrowheads. Scale bars: 20 µm (upper panels), 10 µm (lower panels).

(C) Quantification of STAU2 puncta density in **B**. Data are presented as mean ± SEM (n = 34 [STAU2], n = 34 [STAU2 ΔRBD2], n = 32 [STAU2 4YE]), $***p < 0.001$, Student's *t*-test.

(D) Quantification of the dendrite-to-soma ratio of *CaMKIIα-MS2* in **B**. Data are presented as mean ± SEM (n = 35 [Control], n = 35 [STAU2], n = 36 [STAU2 ΔRBD2], n = 39 [STAU2 4YE]), $**p < 0.01$, $***p < 0.001$, Student's *t*-test.

(E) Quantification of the relative amount of dual-positive ($STAU2^+$/$CaMKII\alpha^+$) puncta in **B**. Data are presented as mean ± SEM (n = 34 [STAU2], n = 34 [STAU2 ΔRBD2], n = 32 [STAU2 4YE]), $***p < 0.001$, Student's *t*-test.

(F) Representative kymographs of *CaMKIIα-MS2* granules in the dendrites of DIV10 rat hippocampal neurons transfected with the indicated constructs, with mobile *CaMKIIα-MS2* granules indicated by red arrowheads. Scale bars: x = 10 µm, y = 5 min.

(G) On DIV9, rat hippocampal neurons were transfected with the *CaMKIIα-MS2* and MCP-SNAP mRNA reporter system along with GFP-N1, GFP-STAU2, GFP-STAU2 ΔRBD2, or GFP-STAU2 4YE constructs. Live-cell imaging of both GFP and *CaMKIIα-MS2* was performed using a spinning disc confocal microscope on DIV10. Representative images show the distribution of GFP-STAU2 (upper panels) and *CaMKIIα-MS2* (lower panels) in dendrites of hippocampal neurons. Red triangles indicate *CaMKIIα-MS2* granules in distal dendrites. Scale bars: 10 µm.

(H) Quantification of *CaMKIIα-MS2* levels in distal dendrites from **G**. Data are presented as mean ± SEM (n = 45 [GFP], n = 61 [STAU2], n = 50 [STAU2 ΔRBD2], n = 53 [STAU2 4YE]), $***p < 0.001$, Student's *t*-test.

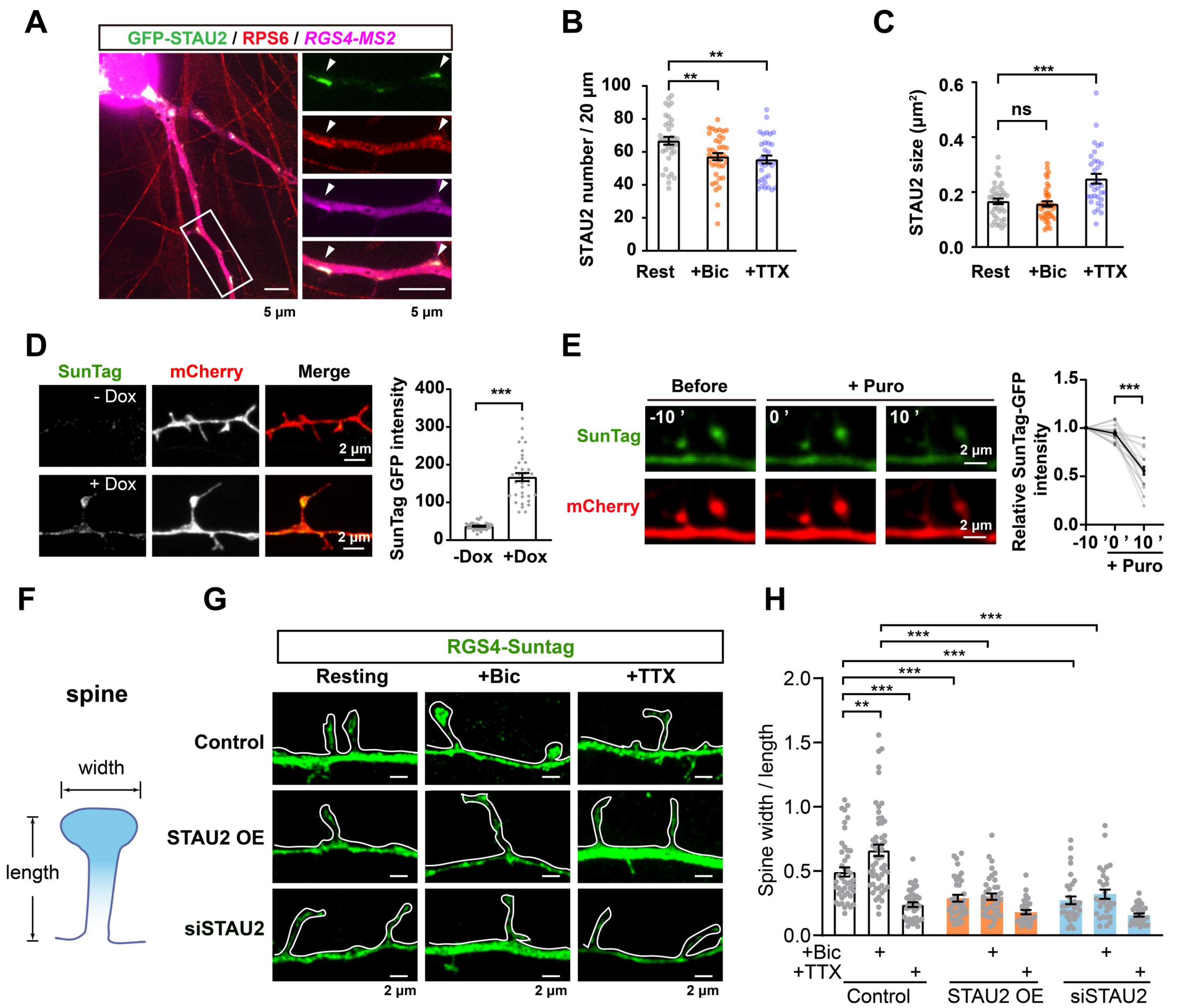

Figure S4. Synaptic activity regulates the assembly pattern of STAU2 granules in dendritic shafts and spines.

(A) DIV9 rat hippocampal neurons were co-transfected with the RGS4-MS2 and MCP-SNAP mRNA reporter systems along with GFP-STAU2. On DIV10, cells were fixed and immunostained for the endogenous ribosomal marker RPS6 (red). The boxed region is enlarged in the right panels. Arrowheads indicate GFP-STAU2 granules that colocalize with ribosomes. Scale bars, 5 µm.

(B, C) Quantification of endogenous STAU2 granule assembly in dendrites, showing the number **(B)** and size **(C)** of STAU2 puncta under resting, +Bic, and +TTX conditions. Data are mean ± SEM (n = 38 [Rest]; n = 40 [+Bic]; n = 33 [+TTX]), $^{**}p < 0.01$, $^{***}p < 0.001$, Student's *t*-test.

(D) Left: Representative confocal images of dendrites from DIV14 rat hippocampal neurons co-expressing the SunTag reporter and mCherry vector, treated with (+Doxy) or without (-Doxy) doxycycline to induce RGS4 peptide synthesis. Scale bars, 2 µm. **Right:** Quantification of SunTag/ScFv-GFP intensity in dendritic spines under the conditions shown on the **left**. Data are mean ± SEM (n = 36 for each group), $^{***}p < 0.001$, Student's *t*-test.

(E) On DIV13, rat hippocampal neurons were co-transfected with the SunTag reporter and mCherry vector. On DIV14, neurons were treated with doxycycline for 4 h to induce RGS4 peptide synthesis prior to live-cell imaging. SunTag signals were acquired by spinning-disk confocal microscopy for 10 min, followed by addition of puromycin to inhibit translation and continued imaging for another 10 min. **Left:** Representative confocal images showing SunTag signals in dendrites from DIV14 rat

hippocampal neurons. The boxed region is magnified in the right panels as a time-lapse series. Scale bars, 5 µm (left), 2 µm (right). **Right:** Quantification of relative SunTag/ScFv-GFP intensity in dendritic spines under the conditions shown on the **left.** Paired spots represent GFP intensity of the same spine before and after puromycin treatment.

(F) Schematic illustrating the dendritic spine width-to-length ratio used for morphological analysis.

(G) Representative confocal images of dendrites from DIV14 rat hippocampal neurons co-transfected with the RGS4-SunTag and either empty vector (Control), mCherry-STAU2 (STAU2 OE), or STAU2 siRNA (siSTAU2) under the three synaptic conditions described above. Spine morphology was outlined. Scale bar, 2 µm.

(H) Quantification of dendritic spine morphology, presented as the dendritic spine width-to-length ratio. Data represent means ± SEM (n= 45, 54, 40, 36, 38, 32, 33, 30 and 30 spines from left to right), ***p< 0.001, One-way ANOVA.

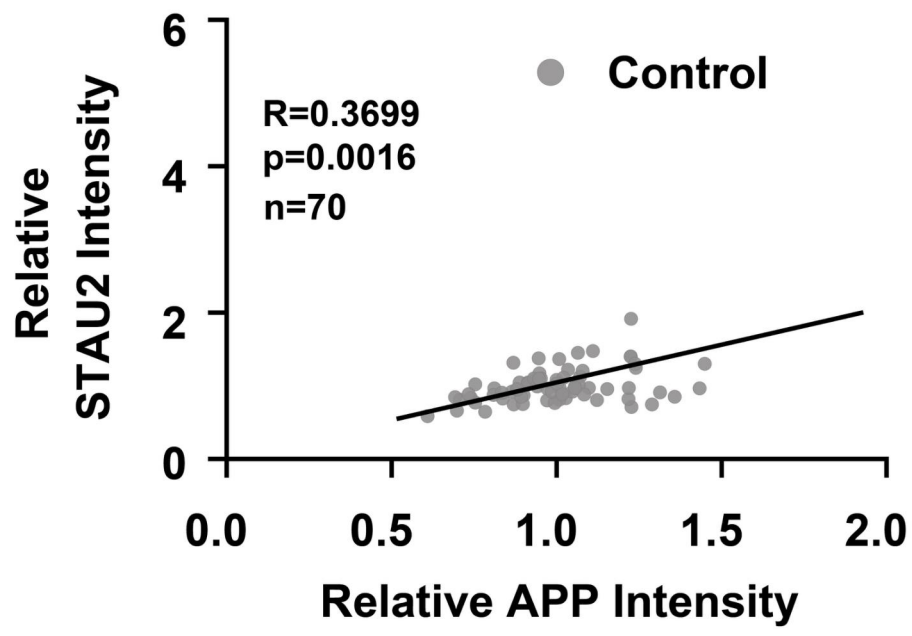

Figure S5. STAU2 weakly correlates with APP levels in hippocampal neurons of wild type mice. Scatter plot of endogenous STAU2 intensity versus APP intensity in hippocampal CA3 neurons from age-matched wild-type mice. Linear regression (black line) reveals a weak positive correlation (Pearson's r = 0.37, p = 0.0016; n = 70 neurons from 3 mice).
